# Dietary Tryptophan Supplementation Alters Fat and Glucose Metabolism in a Low-Birthweight Piglet Model

**DOI:** 10.3390/nu13082561

**Published:** 2021-07-26

**Authors:** Parniyan Goodarzi, Mohammad Habibi, Kennedy Roberts, Julia Sutton, Cedrick Ndhumba Shili, Dingbo Lin, Adel Pezeshki

**Affiliations:** 1 Department of Animal and Food Sciences, Oklahoma State University, Stillwater, OK 74078, USA; parniyan.goodarzi@okstate.edu (P.G.); mohammad.habibi@okstate.edu (M.H.); kennedy.roberts@okstate.edu (K.R.); julia.sutton@okstate.edu (J.S.); cedrick.shili@okstate.edu (C.N.S.); 2 Department of Nutritional Sciences, Oklahoma State University, Stillwater, OK 74078, USA; dingbo.lin@okstate.edu

**Keywords:** tryptophan, glucose and fat metabolism, 5-HT3 receptors, low birthweight, piglet

## Abstract

Low birthweight (LBW) is associated with metabolic complications, such as glucose and lipid metabolism disturbances in early life. The objective of this study was to assess: (1) the effect of dietary tryptophan (Trp) on glucose and fat metabolism in an LBW piglet model, and (2) the role peripheral 5-hydroxytryptamine type 3 (5HT3) receptors in regulating the feeding behavior in LBW piglets fed with Trp-supplemented diets. Seven-day-old piglets were assigned to 4 treatments: normal birthweight-0%Trp (NBW-T0), LBW-0%Trp (LBW-T0), LBW-0.4%Trp (LBW-T0.4), and LBW-0.8%Trp (LBW-T0.8) for 3 weeks. Compared to LBW-T0, the blood glucose was decreased in LBW-T0.8 at 60 min following the meal test, and the triglycerides were lower in LBW-T0.4 and LBW-T0.8. Relative to LBW-T0, LBW-T0.8 had a lower transcript and protein abundance of hepatic glucose transporter-2, a higher mRNA abundance of glucokinase, and a lower transcript of phosphoenolpyruvate carboxykinase. LBW-T0.4 tended to have a lower protein abundance of sodium-glucose co-transporter 1 in the jejunum. In comparison with LBW-T0, LBW-T0.4 and LBW-T0.8 had a lower transcript of hepatic acetyl-CoA carboxylase, and LBW-T0.4 had a higher transcript of 3-hydroxyacyl-CoA dehydrogenase. Blocking 5-HT3 receptors with ondansetron reduced the feed intake in all groups, with a transient effect on LBW-T0, but more persistent effect on LBW-T0.8 and NBW-T0. In conclusion, Trp supplementation reduced the hepatic lipogenesis and gluconeogenesis, but increased the glycolysis in LBW piglets. Peripheral serotonin is likely involved in the regulation of feeding behavior, particularly in LBW piglets fed diets supplemented with a higher dose of Trp.

## 1. Introduction

Newborns who fail to reach an estimated fetal biometric by a certain gestational age (i.e., below the 10th percentile for gestational age) are considered small for gestational age [1]. The incidence of low-birthweight (LBW) infants, weighing less than 2500 g at birth, is 8.31% in the United States, with one third of these infants being recognized as intrauterine growth retarded (IUGR) [2,3,4]. The LBW is not only associated with a higher risk of mortality and morbidity and the development of complications, such as insulin resistance, cardiovascular disease, hypertension, adverse lipid metabolism, dyslipidemia, and obesity in the long term [5,6,7,8], but also related with metabolic disturbances of glucose and lipid metabolism in the short term [9,10]. IUGR rats show alterations in the expression of genes involved in hepatic energy production [11] and exhibit hyperglycemia due to an increased gluconeogenesis as a result of impaired mitochondrial oxidative phosphorylation and pyruvate oxidation in the liver [12]. Similarly, LBW infants [13] and IUGR pigs [14,15] develop hyperglycemia in early life. In addition to impairment in glucose metabolism, the utilization of circulating triglycerides is compromised in LBW infants [16]. Further, a lower insulin sensitivity and less optimized lipid metabolism in early postnatal life have been reported in LBW infants [9]. Similarly, a low activity for hepatic lipoprotein lipase has been shown in IUGR piglets in comparison to their normal-weight counterparts [17]. Therefore, there is evidence that glucose and lipid metabolism is impaired in LBW neonates during early life, which may ultimately lead to the occurrence of metabolic disorders in the short term and long term. Hence, efficient strategies need to be developed to improve the glucose and lipid metabolism in LBW infants during the early postnatal period.

Nutrition is one of the important factors that can influence the glucose and lipid metabolism during the fetal and postnatal periods [8]. In particular, early postnatal nutrition is crucial for the optimal growth [8] and health of LBW infants. Further research is warranted to identify the role of supplemental amino acids (AA), which can improve the growth and glucose and lipid metabolism in LBW infants. Due to limitations regarding the ethical use of LBW infants for nutritional research, animal models are used to study the effect of nutritional interventions on metabolism in LBW offspring. LBW neonatal pigs have significant similarities with LBW infants in the structure and function of the gastrointestinal tract and nutrient metabolism and hence are used as models for LBW infants [18,19,20,21].

Tryptophan (Trp) is a neutral essential amino acid, which, due to its role in the regulation of various physiological functions, may potentially be used for developing therapeutics for preventing and treating chronic diseases [22]. Dietary Trp supplementation has been shown to reduce the serum lipids in rats [23] and the hepatic lipids in laying hens and broilers [24,25]. Further, dietary Trp suppress the hyperglycemia in rodents [22,26]. To our knowledge, no study has assessed the glucose and lipid metabolism of LBW neonates fed with milk-based diets supplemented with different doses of Trp. Trp, as a precursor of central serotonin, has been shown to have anorexigenic effects [27,28,29,30]. However, little is known on the role of peripheral serotonin in the regulation of feeding behavior. The effects of serotonin on appetite regulation are mediated by multiple receptor subtypes, including 5-hydroxytryptamine type 3 (5HT3) receptors [28,31]. It is unknown whether peripheral 5HT3 receptors are essential for feed intake regulation in LBW pigs fed with Trp-enriched diets. Therefore, the objective of this study was to investigate: (1) the effect of two different doses of dietary Trp on blood glucose and triglyceride concentrations and the mRNA and protein expression of key markers related to lipid and glucose metabolism in an LBW piglet model, and (2) the role peripheral 5HT3 signaling in regulating the feeding behavior in LBW piglets fed with Trp-supplemented diets. Further, the effect of supplemental Trp on feed intake, growth measurements, thermal radiation, plasma metabolites, gut development, and hormones was assessed.

## 2. Materials and Methods

### 2.1. Animals and Housing

The experimental procedures used in this study were in accordance with the FASS Guide for the Care and Use of Agricultural Animals in Research and Teaching [32] and were approved by Oklahoma State University’s Institutional Animal Care and Use Committee (IACUC; Animal Care and Use Protocol-IACUC-19-71). Seven-day-old male piglets were selected from twelve sows (Duroc sire line and Large White X Landrace dam; Seaboard, Hennessey, OK, USA) with a similar range of parity (2–4) and litter size (14–18). All piglets received the intramuscular injection of iron dextran complex (200 mg/mL) on day 3 postpartum (1 mL; 100 mg/kg body weight). The piglets were individually housed in plastic floor pens (0.86 × 0.79 × 0.81 m), equipped with milk replacer drinkers and a heat mat. The lighting program was based on a 16:8 h light: dark cycle [33], with lights on at 0900 and off at 0100 during the first week and then lights on at 0800 and off at 0000 in weeks 2 and 3 of the study, respectively. The room temperature was set at 30, 29, and 28 °C during week 1, 2, and 3 of the study, respectively.

### 2.2. Experimental Design and Diets

**Figure 1:**
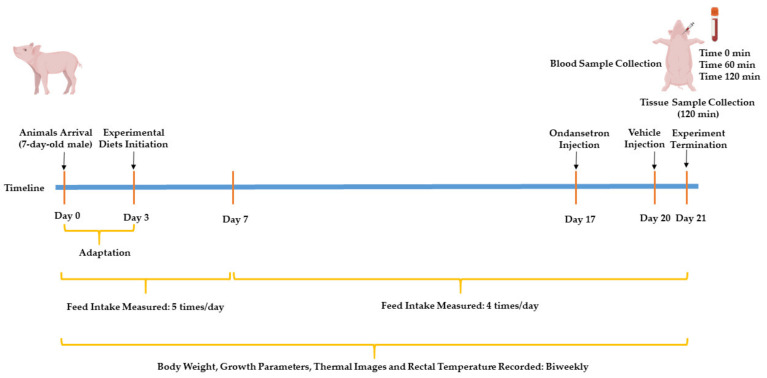
The experimental timeline. Seven-day-old male piglets arrived on day zero and were assigned to low birthweight (LBW) or normal birthweight (NBW) groups, according to their body weight. Following three days of adaptation, LBW pigs were weight-matched, and then NBW and LBW pigs were subjected to one of four dietary treatments, including: NBW-T0, normal-birthweight piglets fed a basal diet without supplemented L-tryptophan (Trp); LBW-T0, low-birthweight piglets fed a basal diet without supplemented Trp; LBW-T0.4, low-birthweight piglets fed a basal diet supplemented with 0.4% Trp; and LBW-T0.8, low-birthweight piglets fed a basal diet supplemented with 0.8% Trp. Following overnight fasting, all pigs received injections of ondansetron and saline on day 17 and 20, respectively. The feed intake was measured 5 times/day during the first week and 4 times/day during the last two weeks of the study. The body weight, growth parameters, thermal images, and rectal temperature were recorded biweekly throughout the study. After an overnight fast at week three, the pigs were allowed to consume their respective diets for 1 h (meal test). Blood samples were then collected at baseline and then at 60 and 120 min after the meal test. Following blood collection at 120 min after the meal test, all pigs were euthanized, and tissue samples were collected.

The body weight (BW) at day 7 postpartum was used as a criterion for considering pigs as LBW or normal birthweight (NBW). Seven-day-old piglets with a BW < 2 kg were considered as LBW, and piglets with a BW ≥ 2 kg were considered as NBW, in accordance with previous studies [34,35]. NBW was included in the experimental design as a control for the LBW animal model used, regardless of the levels of Trp supplemented. The average BW of LBW and NBW piglets at arrival (i.e., day 7 after birth) were 1.66 ± 0.24 and 2.75 ± 0.47 kg, respectively. Prior to starting the experimental diets, following 3 days of adaptation, one NBW and three LBW piglets from each litter were assigned to one of following treatments, while maintaining the mean BW consistent for LBW pigs (2.93 ± 0.49 kg): (1) NBW-T0 (4.39 ± 0.45 kg, n = 8), NBW piglets fed a basal diet without supplemented Trp; (2) LBW-T0 (n = 8), LBW piglets fed a basal diet without supplemented Trp; (3) LBW-T0.4 (n = 7), LBW piglets fed a basal diet supplemented with 0.4% Trp; and (4) LBW-T0.8 (n = 8), LBW piglets fed a basal diet supplemented with 0.8% Trp for 3 weeks (Supplemental Table S1). The experimental timeline is illustrated in Figure 1.

Milk replacer powder was formulated for piglets (Supplemental Table S1), according to the nutrient requirements suggested by previous publications for neonatal pigs [36,37]. For the diet formulations, the chemical composition of the analyzed ingredients in this study (i.e., Arg, His, Phe, whey powder, and whey protein concentrate; Supplemental Tables S2 and S3) and our previous study was used [38]. Except for L-Trp, the amount of ingredients used across all three diets were kept consistent, and all diets were prepared as isonitrogenous and isocaloric by adding L-alanine and dextrose, respectively. A liquid diet was prepared by mixing 1 kg of milk replacer powder (dry matter: 95.6%) with 4.15 L of warm water (40 °C) to obtain a milk replacer with a similar dry matter to sow’s milk, with 18.6% dry matter [39]. Fresh liquid milk replacers were prepared daily and stored at 4 °C throughout the day. Prior to feeding, the milk replacers were transferred to plastic bottles, warmed in a water bath (40 °C), and then offered to pigs in milk replacer feeders. Liquid milk was offered 5 times/day at 0900, 1400, 1900, 0000, and 0500 during the first week and then 4 times per day at 0800, 1400, 2000, and 0200 during weeks 2 and 3 of the study (Figure 1). The piglets had *ad-libitum* access to their diets during each meal and were provided a minimum 60 g dry matter/kg BW/day of milk replacer, as previously suggested [39,40].

#### 2.2.1. Feed Intake and Growth Measurements

The milk replacer intake for each offering was measured by recording the volume of added milk to feeders and the leftover milk in the feeders, prior to the next feeding. Body weight and growth measurements, including heart girth, body length, and wither size, were recorded biweekly. The average daily gain (ADG; gained weight divided by experimental days), average dry matter intake (ADMI; consumed dry matter during feeding period divided by experimental days), average daily protein intake (ADPI; analyzed CP% × dry matter intake (DMI)/experimental days), gain:feed ratio (G:F; ADG divided by ADMI), and gain:protein ratio (G:P; ADG divided by ADPI) were calculated.

#### 2.2.2. Thermal Images and Rectal Temperature

Individual thermal images were captured biweekly using a FLIR C2 compact thermal camera, with focal length of 1.54 mm and thermal accuracy of ±2 °C (FLIR Systems, Boston, MA, USA). The camera was positioned roughly 1 m above of each pig. The emissivity coefficient was set at 0.96. To measure the piglets’ core body temperature, the rectal temperatures (RT) were measured biweekly for all piglets using an electronic thermometer.

#### 2.2.3. Blockade of 5-hydroxytryptamine Type 3 (5HT3) Receptors 

The role of 5HT3 receptors in mediating the effect of experimental diets on the DMI of the pigs was evaluated by the administration of ondansetron hydrochloride (Tocris, Burlington, NA, USA, #2891), a selective 5-HT3 receptor antagonist, and vehicle (0.9% saline). Following an overnight fast, ondansetron (10 mL; 200 μg/kg in sterile 0.9% saline) [41] and saline (10 mL) were injected into all pigs subcutaneously on days 17 and 20 of the study, respectively at 0700 (Figure 1), followed by feeding and recording of the DMI at 0800, 1100, 1400, 1700, 2000, 0200 (next day), and 0800 (next day).

#### 2.2.4. Feed Samples Collection

During the diet preparations, the feed samples (~50 g) were collected from each feed bag and combined for each diet. Afterwards, they were stored at −20 °C, until feed composition analysis.

#### 2.2.5. Meal Test and Blood and Tissue Collection

After an overnight fast (8 h) at week 3, the pigs were allowed to consume their respective diets for 1 h. Blood samples were collected at baseline and then at 60 and 120 min after the meal test from the jugular vein in the supine position into 10 mL serum tubes and 3 mL heparin containing plasma tubes (BD Vacutainer^®^, Franklin Lakes, NJ, USA). The blood samples were centrifuged at 3000× *g* for 15 min at 4 °C, and the serum and plasma were separated and stored at −80 °C for further analysis. Following the blood collection at 120 min after the meal test, all pigs were euthanized using the CO_2_ asphyxiation method. As previously described [38], the hypothalamus, duodenum, jejunum, white adipose tissue, and liver were collected immediately after euthanasia, flushed with distilled water, snap-frozen in liquid nitrogen, and stored at −80 °C for further analyses.

### 2.3. Diet Proximate and Amino Acids Analysis

The dry matter, crude protein, crude fiber, calcium, and phosphorus of the diets and some ingredients contents (i.e., Arg, His, Phe, and whey powder) were analyzed by Servi-Tech laboratory (Dodge City, KS, USA) (Supplemental Table S2), as previously described [38,42,43,44]. The complete amino acids profile of the experimental diets and whey protein concentrate were analyzed [45] by Agricultural Experiment Station Chemical Laboratories (University of Missouri-Columbia, MO, USA) (Supplemental Tables S3 and S4).

### 2.4. Thermal Radiation Analysis

Using a free drawing tool of the FLIR camera software (FLIR Research Studio software), a region of interest was drawn in a rectangular shape on the entire back of the piglets, roughly from shoulders to the rump of the animal (Supplemental Figure S1), to obtain the dorsal surface body average temperature. The heat loss by thermal radiation (W/m^2^) was determined using the following equation: σε (T_s_^4^ − T_α_^4^), where σ is the Stefan Boltzmann Constant (5.67 × 10^−8^ W/m^2^K^4^), ε is the thermodynamic emissivity (0.95), T_s_ is the body surface temperature (kelvin), and T_α_ is the ambient temperature (kelvin) [46,47].

### 2.5. Plasma Metabolites Analysis

The plasma glucose, triglycerides, and cholesterol concentration were determined by a chemistry analyzer system (CLC 480/BioLis24i, Carolina Liquid Chemistries Corp., Brea, CA), following calibration with a calibrator (Catalogue #: BL-442600, Multi-Analyte calibrator for Synchron CX/LX), using reagents (Carolina Liquid Chemistries Crop, Brea, CA, USA) for glucose (Catalogue #: BL-208), cholesterol (Catalogue #: BL-211), and triglycerides (Catalogue #: BL-213), and recording the absorbance at 340 nm for glucose and at 505 nm for triglycerides and cholesterol.

### 2.6. H&E Staining and Gut Morphology Measurements

Duodenum and jejunum segments were fixed in 10% formaldehyde, coated in paraffin, cut into 5 μm thick sections, and stained with hematoxyl and eosin. Ten well-oriented villi and crypt in each section were used to measure the villus height, villus width, crypt depth, crypt width, and muscle thickness [48] using a BZ-X800E Keyence all-in-one Fluorescence Microscope (BZ-X710, IL, USA), with the images taken with the Keyence BZ-X Viewer (Keyence Co. USA, Itasca, IL, USA) and the analysis performed using the ImageJ software (2 April 2020, https://imagej.nih.gov/ij/download.html).

### 2.7. RNA Isolation and RT-qPCR

RNA isolation and RT-qPCR were performed for glucose transporter 1 (GLUT1), glucokinase (GCK), liver-type phosphofructokinase (PFKL), liver-type pyruvate kinase (PKLR), pyruvate carboxylase (PC), phosphoenolpyruvate carboxykinase (PEPCK), glucose-6-phosphatase (G6PC), glucose transporter 2 (GLUT2), lipoprotein lipase (LPL), cluster of differentiation 36 molecule (CD36), fatty acid synthase (FAS), acetyl-CoA carboxylase (ACC), hydroxyacyl-CoA dehydrogenase (HADH), sterol regulatory element binding transcription factor 1 (SREBP-1), peroxisome proliferator activated receptor alpha (PPARα), and PPARG coactivator 1 alpha (PGC1α) in the liver, following our published procedures [38,49,50]. The quality of isolated RNA was measured by a NanoDrop ND-1000 spectrophotometer (Thermo Fisher, Waltham, MA, USA). The complementary cDNA was synthesized by a thermoscycler (T100TM Thermal Cycler, Bio-Rad, Hercules, CA, USA), and then a CFX96 real-time PCR detection system (Bio-Rad, Hercules, CA, USA) was used to measure the mRNA abundance of the target genes through real-time quantitative PCR (qPCR), followed by a melt curve, as previously described [38,49,50]. The primers used are shown in Supplemental Table S5 and were obtained from previous publications [51,52,53,54,55,56,57,58]. β-actin was used as a housekeeping gene, and the relative mRNA abundance of the target genes was calculated by the 2^−∆∆CT^ method.

### 2.8. Immunoblot Analysis

Western blot was conducted in hypothalamus for tryptophan hydroxylase 2 (TPH2), jejunum for TPH2, sodium/glucose cotransporter 1 (SGLT1), and glucose transporter 2 (GLUT2), duodenum for SGLT1 and GLUT2, liver for carnitine palmitoyltransferase I α (CPT1α) and GLUT2, and white adipose tissues for CPT1α (Supplemental Table S6), as previously described [59,60]. The protein bands were captured using a ChemiDoc XR imaging system (Bio-Rad Laboratories Inc., CA, USA). Image Lab software (Version 6.0.1, Bio-Rad Laboratories Inc., CA, USA) was used for applying densitometry. In order to determine the relative amount of protein abundance in the sample, glyceraldehyde-3-phosphate dehydrogenase (GAPDH) was used as a loading control.

### 2.9. Plasma Insulin

The plasma insulin concentration was determined using a porcine ELISA kit, according to the manufacturer’s instructions (Mercodia Porcine Insulin ELISA; Mercodia AB; Uppsala, Sweden). The optical density was read at a 450 nm wavelength using an Epoch microplate spectrophotometer (BioTek^®^ Instruments, Inc. Highland Park, VT). The intra-assay coefficient of variation (CV) was 4.8%. The insulin resistance indicator, HOMA-IR, was calculated, as previously described [61]. The baseline blood glucose concentrations (G0, milligrams per deciliter) and the fasting blood insulin concentrations (I0, micro-international units per milliliter) were used to calculate HOMA-IR, using the following formula: (G0 × I0)/2430.

### 2.10. Statistical Analysis

The final body weight, heart girth, body length, wither size, ADG, ADMI, ADPI, G:F, G:P, plasma cholesterol and triglycerides, gut histomorphometry, qPCR, ELISA, and western blot data were analyzed by the GLM procedure (IBM SPSS Statistics Version 23, Armonk, NY, USA), followed by a post hoc Dunnett’s test (IBM SPSS Statistics Version 23, Armonk, NY, USA). The plasma glucose concentration distribution was not normal at 60 min after the meal. Therefore, an inverse distribution function (IDF-normal) was used to normalize the data at this time point. The mixed procedure was used for the analysis of blood glucose, DMI, BW, rectal temperature, and thermal radiation, with diet, time, and the interaction of diet and time as fixed variables and pigs as a random variable. As previously described [42,43,50], the covariance structure of repeated measurements was modeled for each variable based on the smallest levels of fit statistics for corrected Akaike’s Information Criterion and Bayesian Information Criterion. Before statistical analysis of all the data, the outlier test was performed in SPSS (IBM SPSS Statistics Version 23, Armonk, NY, USA), which is based on the Interquartile Rule. *p* ≤ 0.05 and 0.05 < *p* ≤ 0.10 were considered as significant and trend, respectively.

## 3. Results

### 3.1. Feed Intake, Body Weight, and Growth Measurements

Overall, except for the G:F ratio, the effect of diet on the initial BW, final BW, ADG, ADMI, ADPI, G:P ratio, heart girth, wither height, and body length was significant (Table 1). NBW-T0 had a greater initial BW, final BW, ADG, ADMI, ADPI, heart girth, wither height, and body length, compared to LBW-T0 (~54, 30, 22, 31, 27, 10, 8, and 11%, respectively). Moreover, ADPI was lower in LBW-T0.8 (11.7%), compared to LBW-T0.

**Figure 2:**
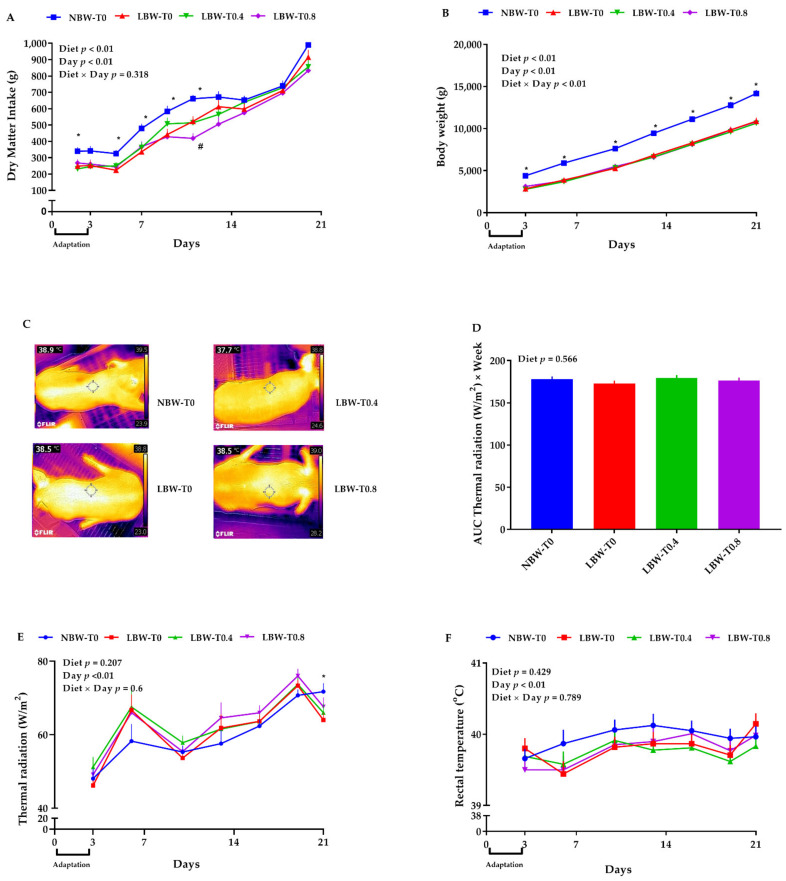
Effect of dietary L-tryptophan supplementation on dry matter intake, body weight, thermal radiation, and area under the curve (AUC) for the thermal radiation and rectal temperature of low-birthweight neonatal pigs. (**A**) dry matter intake, (**B**) body weight, (**C**) representative thermal images, (**D**) area under the curve (AUC) thermal radiation, (**E**) thermal radiation, and (**F**) rectal temperature. NBW-T0, normal-birthweight piglets fed a basal diet without supplemented L-tryptophan (Trp); LBW-T0, low-birthweight piglets fed a basal diet without supplemented Trp; LBW-T0.4, low-birthweight piglets fed a diet supplemented with 0.4% Trp; and LBW-T0.8, low-birthweight piglets fed a diet supplemented with 0.8% Trp. n = 8 for NBW-T0, LBW-T0, and LBW-T0.8, and n = 7 for LBW-T0.4. * *p* ≤ 0.05 vs. LBW-T0; ^#^ 0.05 < *p* ≤ 0.1 vs. LBW-T0. The values are the means ± SEM.

Overall, the effect of diet and day on daily DMI was significant (Figure 2A). Compared to LBW-T0, NBW-T0 had a higher (~26–45%) DMI on days 2, 5, 7, 9, and 11 of the study (Figure 2A). LBW-T0.8 tended to have a lower DMI, compared to LBW-T0, on day 11 (Figure 2A). The 1-h meal test DMI for NBW-T0, LBW-T0, LBW-T0.4, and LBW-T0.8 was 154 ± 10 g, 126 ± 19 g, 125 ± 18 g, and 123 ± 17 g, respectively. As expected, the DMI of NBW-T0 vs. LBW-T0 was significantly different (*p* < 0.05), but no differences in DMI were seen among the other groups. The effect of diet on BW was significant, with 9310, 6838, 6687, and 6787 g for NBW-T0, LBW-T0, LBW-T0.4, and LBW-T0.8, respectively (Figure 2B). Compared to LBW-T0, NBW-T0 had a higher BW throughout the study (~30–54%). No differences in the BW of pigs were detected, when LBW-T0 was compared with LBW-T0.4 or LBW-T0.8 (Figure 2B).

### 3.2. Thermal Radiation

Compared to LBW-T0, no differences in the thermal radiation, the area under the curve (AUC) for thermal radiation, and the rectal temperature were detected among the treatments (Figure 2C–F).

### 3.3. Gut Histomorphology

**Figure 3:**
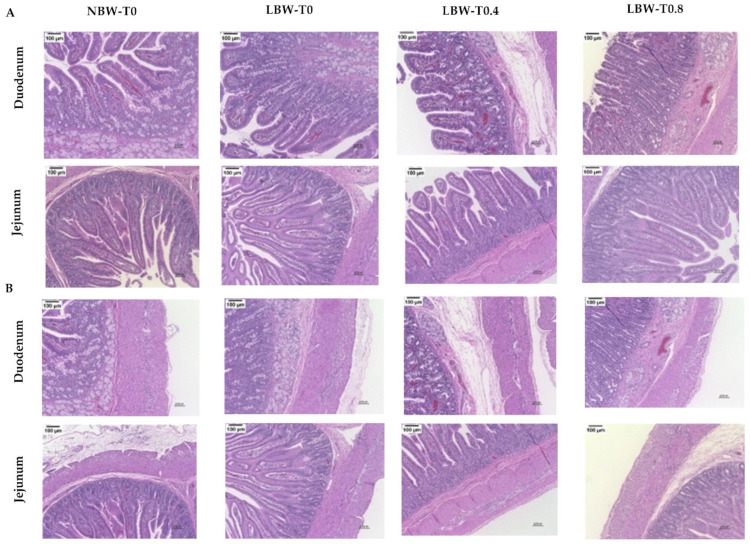
Effect of dietary L-tryptophan supplementation on the morphology and development of the duodenum and jejunum in low-birthweight neonatal pigs. Representative hematoxylin and eosin-stained sections (×10 magnification) micrograph of the duodenum and jejunum for (**A**) villi and (**B**) muscle. NBW-T0, normal-birthweight piglets fed a basal diet without supplemented L-tryptophan (Trp); LBW-T0, low-birthweight piglets fed a basal diet without supplemented Trp; LBW-T0.4, low-birthweight piglets fed a diet supplemented with 0.4% Trp; and LBW-T0.8, low-birthweight piglets fed a diet supplemented with 0.8% Trp.

The histomorphometry data showed a tendency toward a greater villus height to crypt depth ratio in NBW-T0, compared to LBW-T0 in duodenum (Table 2; Figure 3). Compared to LBW-T0, the crypt depth tended to be higher in LBW-T0.8 in jejunum. No differences among the groups were observed for the villus height, villus width, crypt width, and muscle thickness in both duodenum and jejunum (Table 2; Figure 3).

### 3.4. 5HT3 Receptors Blockade with Ondansetron

**Figure 4:**
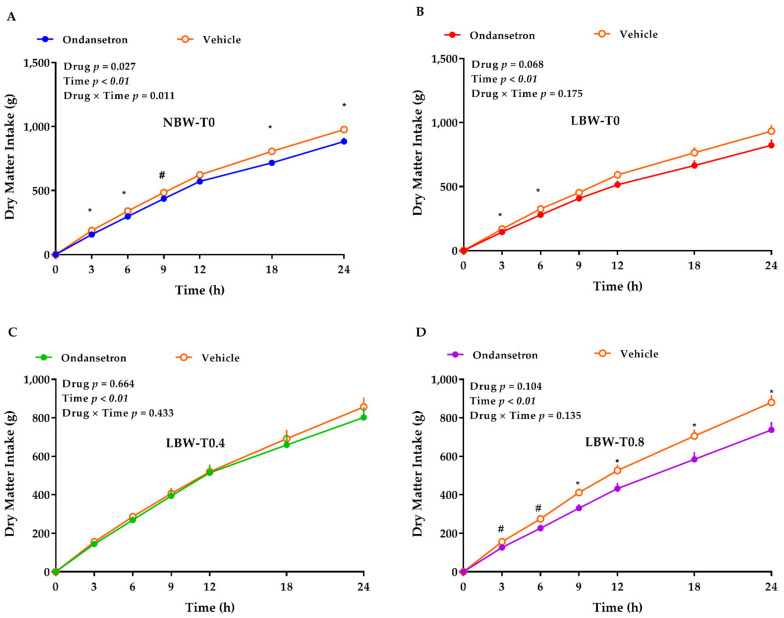
Effect of saline or ondansetron injection on the dry matter intake of low-birthweight neonatal pigs fed with milk-based diets supplemented with L-tryptophan. (**A**) NBW-T0, normal-birthweight piglets fed a basal diet without supplemented L-tryptophan (Trp), (**B**) LBW-T0, low-birthweight piglets fed a basal diet without supplemented Trp, (**C**) LBW-T0.4, low-birthweight piglets fed a diet supplemented with 0.4% Trp, and (**D**) LBW-T0.8, low-birthweight piglets fed a diet supplemented with 0.8% Trp. All pigs were injected either with saline or the drug (ondansetron, 200 μg × kg^−1^, SC). The *p* values for the overall model effect for the drug, time, diet, drug × time, drug × diet, time × diet and drug × time × diet for dry matter intake were <0.01, <0.01, 0.083, <0.01, 0.470, 0.108, and 0.707, respectively. n = 8 for NBW-T0, LBW-T0, and LBW-T0.8, and n = 7 for LBW-T0.4. * *p* ≤ 0.05 saline vs. ondansetron within each diet; ^#^ 0.05 < *p* ≤ 0.1 saline vs. ondansetron within each diet. The values are the means ± SEM.

Overall, the effect of the drug on DMI was significant (*p* < 0.01; Figure 4), showing a lower DMI for ondansetron vs. vehicle (466 ± 14 vs. 518 ± 14 g, respectively), and the overall effect of diet on DMI tended to be significant (*p* = 0.083; Figure 4), showing the lowest DMI for LBW-T0.8. The DMI for NBW-T0, LBW-T0, LBW-T0.4, and LBW-T0.8 were 540 ± 26 g, 505 ± 26 g, 477± 27 g, and 445 ± 26 g, respectively. Relative to the vehicle, the ondansetron decreased the DMI by ~8–16% in NBW-T0 and 16–20% in LBW-T0.8 during a 24 h period (Figure 4A,D), while the ondansetron reduced the DMI by 14% in LBW-T0 only during the first 6 h (Figure 4B). No differences in DMI were detected when the vehicle and ondansetron were compared for LBW-T0.4 (Figure 4C).

### 3.5. Blood Glucose, Triglycerides, Cholesterol, and Insulin

**Figure 5:**
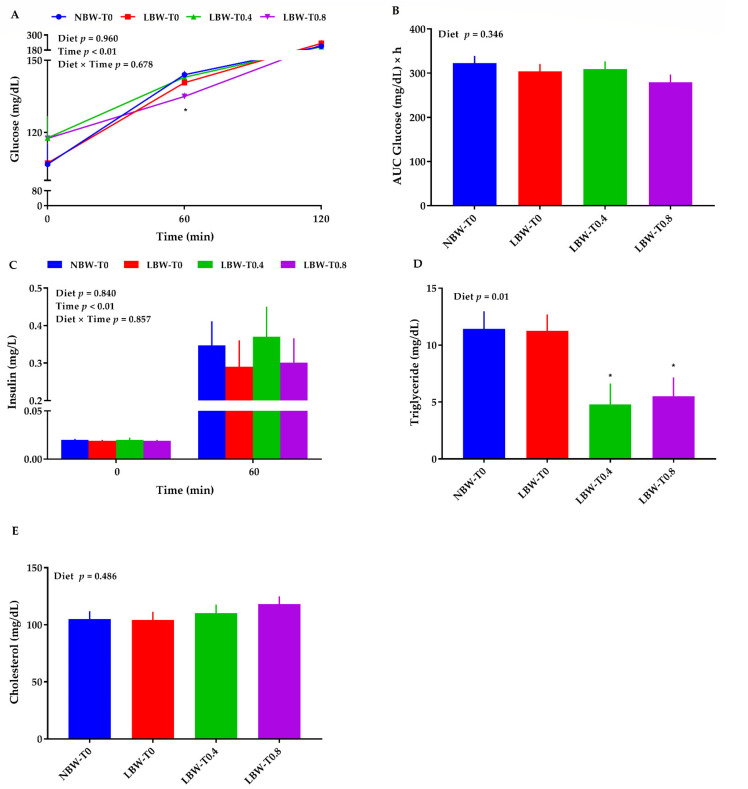
Effect of dietary L-tryptophan supplementation on plasma metabolite concentrations in low-birthweight neonatal pigs. (**A**) glucose, (**B**) area under the curve (AUC) for glucose, (**C**) insulin, (**D**) triglyceride, and (**E**) cholesterol. NBW-T0, normal-birthweight piglets fed a basal diet without supplemented L-tryptophan (Trp); LBW-T0, low-birthweight piglets fed a basal diet without supplemented Trp; LBW-T0.4, low-birthweight piglets fed a diet supplemented with 0.4% Trp; and LBW-T0.8, low-birthweight piglets fed a diet supplemented with 0.8% Trp. n = 8 for NBW-T0, LBW-T0, and LBW-T0.8, and n = 7 for LBW-T0.4. * *p* ≤ 0.05 vs. LBW-T0. The values are the means ± SEM.

No differences in blood glucose were observed among the treatments at 0 and 120 min following the meal test (Figure 5A). However, LBW-T0.8 had a lower (~4%) blood glucose than LBW-T0 at 60 min after the meal challenge (Figure 5A). The AUC for blood glucose did not change across groups (Figure 5B). Likewise, the plasma insulin at 0 and 60 min after the meal challenge (Figure 5C) and HOMA-IR was not different across groups. Relative to LBW-T0, the plasma triglyceride concentration was decreased in LBW-T0.4 and LBW-T0.8 by 57% and 51%, respectively (Figure 5D). The total cholesterol did not differ across groups (Figure 5E).

### 3.6. The mRNA Abundance of Key Molecules of Glucose and Lipid Metabolism in the Liver

**Figure 6:**
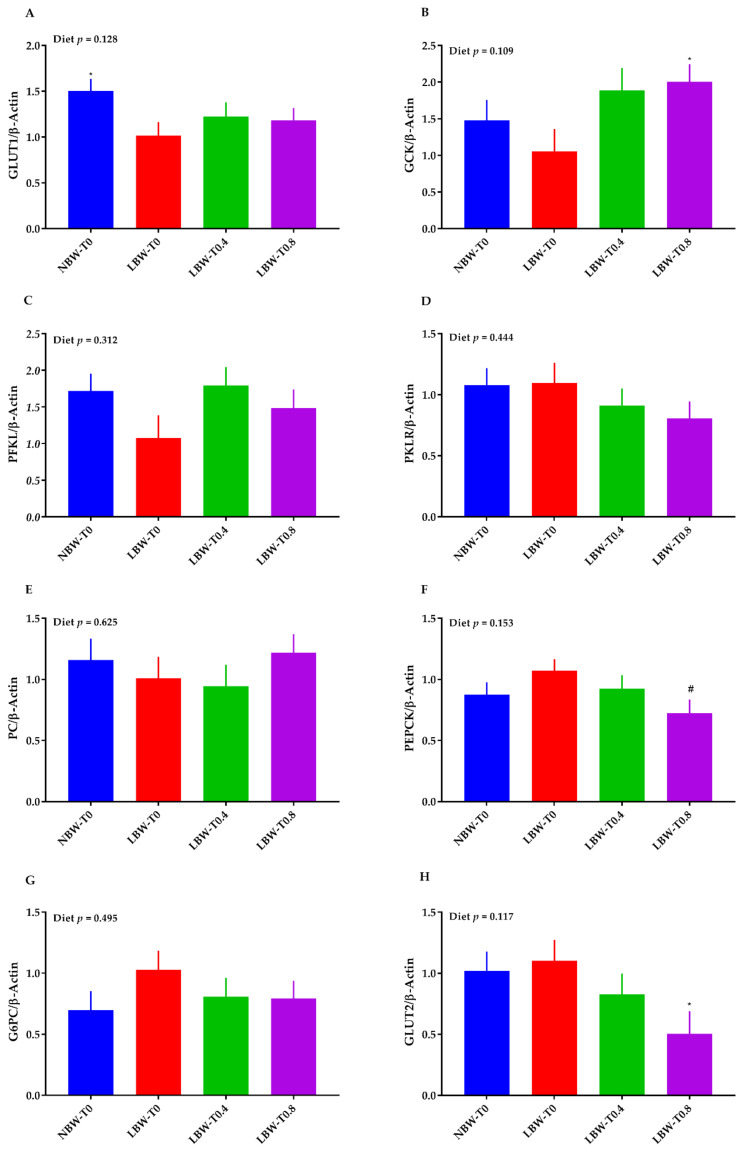
Effect of dietary L-tryptophan supplementation on the liver mRNA abundance of glucose metabolism markers in low-birthweight neonatal pigs. (**A**) glucose transporter 1 (GLUT 1), (**B**) glucokinase (GCK), (**C**) phosphofructokinase, liver-type (PFKL), (**D**) pyruvate kinase, liver, and RBC (PKLR), (**E**) pyruvate carboxylase (PC), (**F**) phosphoenolpyruvate carboxykinase (PEPCK), (**G**) glucose-6-phosphatase catalytic (G6PC), and (**H**) glucose transporter 2 (GLUT2). NBW-T0, normal-birthweight piglets fed a basal diet without supplemented L-tryptophan (Trp); LBW-T0, low-birthweight piglets fed a basal diet without supplemented Trp; LBW-T0.4, low-birthweight piglets fed a diet supplemented with 0.4% Trp; and LBW-T0.8, low-birthweight piglets fed a diet supplemented with 0.8% Trp. n = 8 for NBW-T0, LBW-T0, and LBW-T0.8, and n = 7 for LBW-T0.4. * *p* ≤ 0.05 vs. LBW-T0; ^#^ 0.05 < *p* ≤ 0.1 vs. LBW-T0. The values are the means ± SEM.

Compared to LBW-T0, the mRNA abundance of GLUT1 was higher in NBW-T0 (Figure 6A). Relative to LBW-T0, the mRNA abundance of GCK was greater in LBW-T0.8 (Figure 6B). The mRNA expression of liver GLUT2 was lower, and PEPCK tended to be lower in LBW-T0.8, compared to LBW-T0 (Figure 6H,F). No differences among treatments were detected for the mRNA abundance of PFKL (Figure 6C), PKLR (Figure 6D), PC (Figure 6E), and G6PC (Figure 6G).

**Figure 7:**
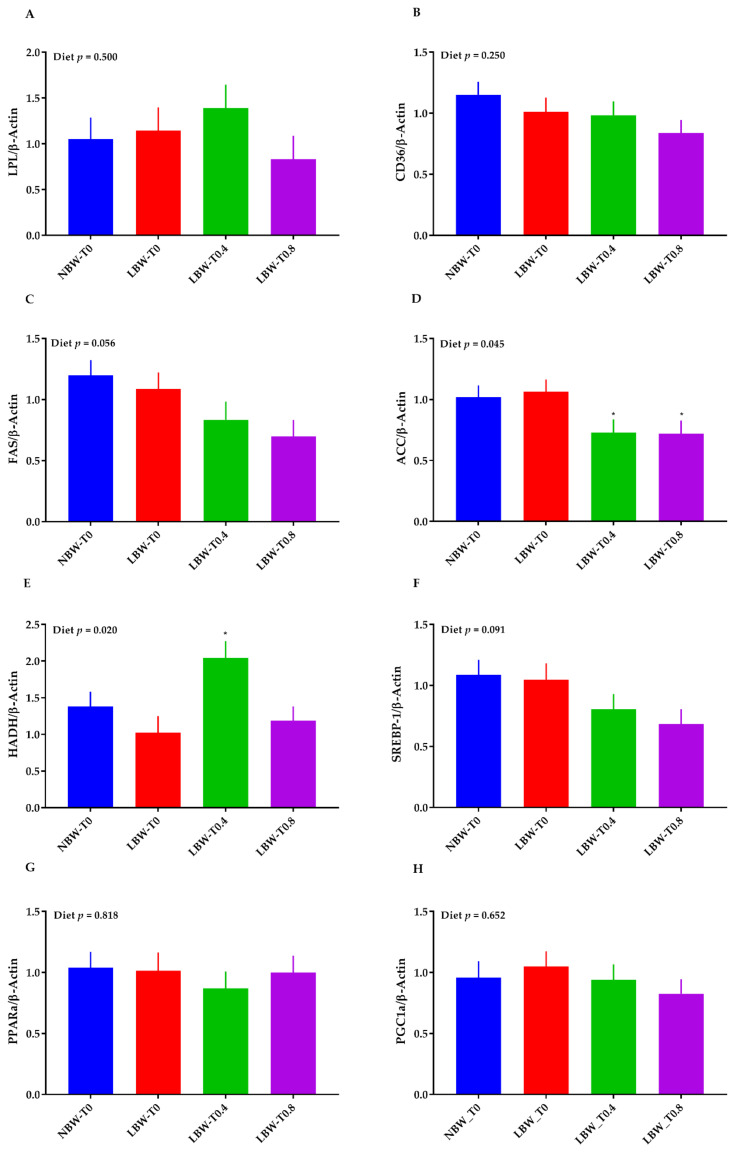
Effect of dietary L-tryptophan supplementation on the liver mRNA abundance of lipid metabolism markers in low-birthweight neonatal pigs. (**A**) lipoprotein lipase (LPL), (**B**) cluster of differentiation 36 molecule (CD36), (**C**) fatty acid synthase (FAS), (**D**) acetyl-CoA carboxylase alpha (ACC), (**E**) hydroxyacyl-CoA dehydrogenase (HADH), (**F**) sterol regulatory element-binding transcription factor 1 (SREBP-1), (**G**) peroxisome proliferator activated receptor alpha (PPARα), and (**H**) PPARG coactivator 1 alpha (PGC1α). NBW-T0, normal-birthweight piglets fed a basal diet without supplemented L-tryptophan (Trp); LBW-T0, low-birthweight piglets fed a basal diet without supplemented Trp; LBW-T0.4, low-birthweight piglets fed a diet supplemented with 0.4% Trp; and LBW-T0.8, low-birthweight piglets fed a diet supplemented with 0.8% Trp. n = 8 for NBW-T0, LBW-T0, and LBW-T0.8, and n = 7 for LBW-T0.4. * *p* ≤ 0.05 vs. LBW-T0. The values are the means ± SEM.

The effect of diet on the mRNA abundance of liver ACC and HADH was significant (*p* < 0.05, Figure 7D,E). The mRNA abundance of ACC was lower in LBW-T0.4 and LBW-T0.8, compared to LBW-T0 (Figure 7D). The transcript of liver HADH was greater in LBW-T0.4, compared to LBW-T0 (Figure 7E). The effect of diet on FAS (Figure 7C) and the SREBP-1 (Figure 7F) transcript tended to be significant, with a lower mRNA abundance for these markers in LBW-T0.4 and LBW-T0.8 than LBW-T0. There were no significant differences across treatments for the mRNA abundance of LPL (Figure 7A), CD36 (Figure 7B), and PPARα (Figure 7G).

### 3.7. The Protein Abundance of Key Molecules of Glucose and Lipid Metabolism in the Hypothalamus, Liver, Jejunum, Duodenum, and White Adipose Tissue

**Figure 8:**
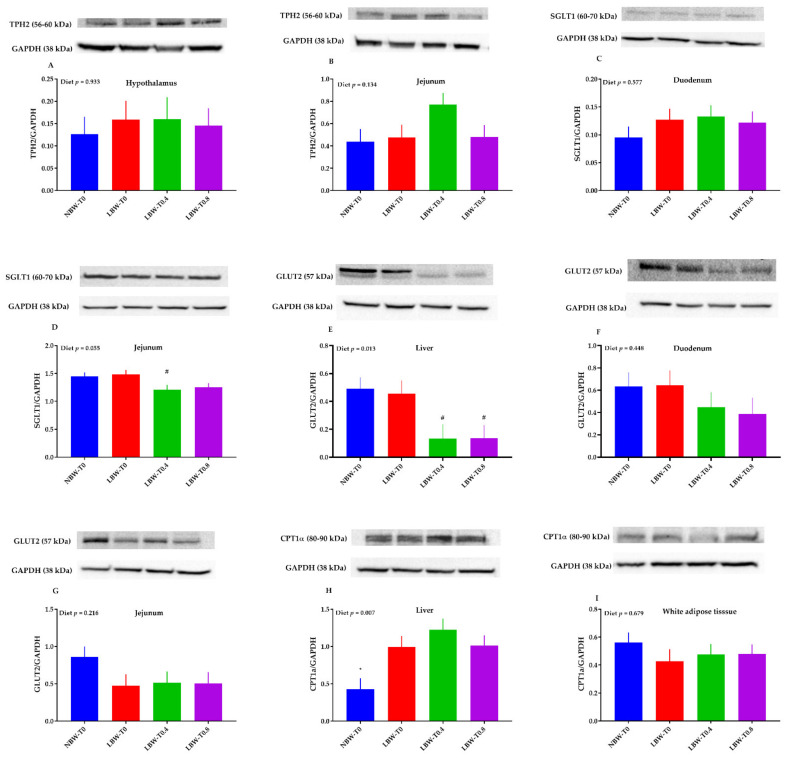
Effect of dietary L-tryptophan supplementation on the relative protein abundance of feed intake and glucose and lipid metabolism markers in the hypothalamus, jejunum, duodenum, liver, and white adipose tissue. (**A**) tryptophan hydroxylase 2 (TPH2) in the hypothalamus, (**B**) TPH2 in the jejunum, (**C**) sodium/glucose cotransporter 1 (SGLT1) in the duodenum, (**D**) SGLT1 in the jejunum, (**E**) glucose transporter-2 (GLUT2) in the liver, (**F**) GLUT2 in the duodenum, (**G**) GLUT2 in the jejunum, (**H**) carnitine palmitoyltransferase I α (CPT1α) in the liver, and (**I**) CPT1α in white adipose tissue. NBW-T0, normal-birthweight piglets fed a basal diet without supplemented L-tryptophan (Trp); LBW-T0, low-birthweight piglets fed a basal diet without supplemented Trp; LBW-T0.4, low-birthweight piglets fed a diet supplemented with 0.4% Trp; and LBW-T0.8, low-birthweight piglets fed a diet supplemented with 0.8% Trp. n = 8 for NBW-T0, LBW-T0, and LBW-T0.8, and n = 7 for LBW-T0.4. * *p* ≤ 0.05 vs. LBW-T0; ^#^ 0.05 < *p* ≤ 0.1 vs. LBW-T0. The values are the means ± SEM.

The protein abundance of SGLT1 tended to be lower in LBW-T0.4, compared to LBW-T0, in the jejunum (Figure 8D). Moreover, relative to LBW-T0, the GLUT2 protein abundance tended to be lower in LBW-T0.4 and LBW-T0.8 in the liver (Figure 8E). Pigs in the NBW-T0 group had a lower protein abundance of CPT1α in the liver than in LBW-T0 (Figure 8H). The protein abundance of TPH2 in the hypothalamus and jejunum (Figure 8A,B), SGLT1 in the duodenum (Figure 8C), GLUT2 in the duodenum and jejunum (Figure 8F,G), and CPT1α in white adipose tissue (Figure 8I) were not different across dietary treatments. The full-length immunoblots for all the above markers are given in Supplemental Figure S2.

## 4. Discussion

LBW is not only associated with health complications in the long term [5,6,7,8], but it is also linked with glucose and lipid metabolism disturbances in the short term [9,10]. Nutrition is crucial for the optimal growth and health of LBW during the fetal and postnatal periods [8]. Dietary Trp decreases the serum and hepatic lipids in rodents and chickens [23,24,25] and suppresses the hyperglycemia in rodents [22,26]. Further, Trp is a precursor of serotonin that is involved in feed intake regulation. Whether dietary Trp affects the glucose and lipid metabolism and influences the feeding behavior through peripheral serotonin in LBW neonates has not been elucidated. The objective of this study was to assess: (1) the effect of dietary Trp on lipid and glucose metabolism in a LBW piglet model, and (2) the role of peripheral 5HT3 receptors on feed intake regulation in LBW pigs fed with Trp-enriched diets. Our study generated several key findings: (1) LBW-T0.8 decreased plasma glucose at 60 min after a meal test, which is indicative of the role of Trp in reducing blood glucose. This might be explained by the increased glycolysis (increased hepatic mRNA abundance of GCK), decreased gluconeogenesis (tending to decrease the PEPCK transcript in the liver), and reduced hepatic glucose efflux (reduced hepatic GLUT2 gene and protein abundance) in the LBW-T0.8 group. (2) The blood glucose concentration did not change in LBW-T0.4, and the protein expression of SGLT1 in the jejunum tended to be decreased and GLUT2 in the liver was decreased in this group, suggestive of a reduced absorption of glucose in the gut and glucose efflux in the liver in this group. (3) Both LBW-T0.4 and LBW-T0.8 reduced the plasma triglyceride concentration. These might be due to the reduced hepatic lipogenesis in both groups (reduced mRNA abundance of hepatic ACC) and increased hepatic lipolysis in LBW-T0.4 (increased mRNA abundance of hepatic HADH). (4) Blocking the 5-HT3 receptor with ondansetron reduced the DMI in all groups, which is suggestive of the possible role of peripheral serotonin in the regulation of feeding behavior. (5) Ondansetron decreased the DMI transiently (6 h by 14%) in LBW-T0, but that reduced the DMI throughout the day (24 h by 16–20%) in LBW-T0.8. Additionally, ondansetron specifically reduced the DMI in the NBW-T0 group. These data are suggestive of a higher serotonergic signaling in the LBW-T0.8 and NBW-T0 groups, compared to LBW-T0. Altogether, milk replacers enriched in Trp improved the hepatic lipid and glucose metabolism by reducing the lipogenesis, gluconeogenesis, and glucose efflux and increasing the lipolysis and glycolysis. Peripheral serotonin is likely involved in the regulation of feeding behavior, particularly in LBW piglets fed diets supplemented with a higher dose of Trp.

To our knowledge, this is the first report on the effect of dietary Trp on glucose metabolism in LBW neonates. The plasma glucose concentration was decreased at 60 min after a meal test in LBW-T0.8. Similarly, others showed that dietary supplementation of Trp reduced hyperglycemia [26] and its injection caused hypoglycemia [62] in rats. Likewise, an intragastric infusion of Trp decreased blood glucose in humans [63], pigs [64], and rats [22]. In contrast, others showed that either the oral administration of Trp in humans [65] or the intragastric administration of Trp in rats [66] increased plasma glucose. Differences in the doses of Trp used, the route of administration (e.g., oral, diet, gastric, and injection), and the experimental units may all lead to discrepancies seen among studies. Intragastric and intravenous tryptophan administration has been reported to produce a differential effect on plasma glucose and hormones controlling blood glucose, such as glucagon and gastric inhibitory polypeptide (GIP) [66]. It appears that the oral or intragastric administration of Trp at supraphysiological levels stimulates glucagon secretion and hyperglycemia [65,66]. Unlike the abovementioned studies, our data suggest a hypoglycemic role for Trp, which might be due to the disposal of glucose by tryptophan by increasing insulin and incretins [67] and glucose-mediated GIP secretion [64], and the physiological effect of Trp in inhibiting the absorption of glucose from the small intestine [26] and slowing gastric emptying [63,68].

Little is understood about the effect of Trp on biochemical pathways involved in glucose metabolism in LBW neonates. To our knowledge, the intestinal absorption of glucose and the dynamics of glucose metabolism in liver, particularly glycolysis and gluconeogenesis, have not been elucidated in LBW neonates receiving Trp-enriched diets. For the first time, here we show an increased hepatic mRNA abundance of GCK, decreased PEPCK transcript in the liver, and reduced hepatic GLUT2 gene and protein abundance in the LBW-T0.8 group. Further, the protein expression of SGLT1 in the jejunum and GLUT2 in the liver was decreased in the LBW-T0.4 group. Unlike our study, where we showed an increased mRNA abundance of GCK in LBW-T0.8, others have either failed to detect a change in the hepatic GCK mRNA expression in rats fed with diets supplemented with Trp [22] or showed a downregulation in the GCK expression in the liver with a higher dose of dietary Trp in blunt snout bream [69]. Differences in the amount of supplemented Trp and animal species used may contribute to the discrepancy in the findings. GCK is one of the key limiting enzymes in glycolysis in the liver, which converts glucose to glucose-6-phosphate, and its expression is regulated by insulin [70]. The plasma insulin did not change across groups in our study; therefore, the increased expression of GCK in response to a high dose of supplemental Trp suggests the existence of an alternative pathway. GCK has three Trp residues in its structure [71], which may explain the link between the availability of Trp and GCK synthesis. The increased transcript of GCK in the current study is suggestive of an increased glycolysis in LBW pigs fed with diets supplemented with 0.8% Trp, which may contribute to the reduced hyperglycemia in this group. In line with our findings, others reported a reduced activity for PEPCK following Trp administration in normal and adrenalectomized rats [72]. However, there are other reports showing an increased PEPCK activity following the administration of Trp in rats [73] and mice [74]. In our study, we did not measure the activity of PEPCK; however, the energy balance status of the animals used (e.g., fed vs. fasted) or possible differences in the regulation of gluconeogenesis by nutrients and amino acids, such as Trp, among different animal species may contribute to the controversial effect of Trp on the PEPCK activity in the abovementioned studies. PEPCK is the first key rate-limiting enzyme in hepatic gluconeogenesis [75]. The lower abundance of PEPCK mRNA in LBW-T0.8 in the current study is suggestive of a reduced gluconeogenesis, which may explain the lower plasma glucose concentration in this group. In the present study, we showed a reduced hepatic GLUT2 expression in both the LBW-T0.4 and LBW-T0.8 groups. To our knowledge, there is no published data on the dynamics of hepatic GLUT2 following Trp administration. The major role of GLUT2 in the liver is glucose efflux following gluconeogenesis, rather than glucose uptake [76]. Therefore, the reduced expression of hepatic GLUT2 in LBW-T0.4 and LBW-T0.8 is indicative of a reduced glucose efflux to the bloodstream through hepatocytes. This may explain the lower blood glucose concentration seen in LBW-T0.8 piglets. Further, we report here a reduced protein abundance of SGLT1 in the jejunum of LBW-T0.4 piglets. Previously, L-Trp was shown to have a strong interaction with SGLT1 [26]. Given the important role of SGLT1 in the absorption of glucose in the gut, our data suggest that Trp inhibits the absorption of glucose through the downregulation of intestinal SGLT1. Overall, the reduced blood glucose in LBW-T0.8 might be explained by the increased glycolysis, decreased gluconeogenesis, and reduced hepatic glucose efflux in the LBW-T0.8 group. While the expression of SGLT1 in the jejunum and GLUT2 in the liver was decreased in the LBW-T0.4 group, the blood glucose did not change in the same group. Unlike the higher dose of Trp (T0.8), which changed the expression glycolytic and gluconeogenic enzymes, the lower dose of supplemental Trp appears to only influence the uptake of glucose from the intestine and efflux of glucose in hepatocytes, which does not seem to be sufficient to change the blood glucose level.

In the current study, for the first time, we show a decreased plasma triglyceride concentration in LBW-T0.4 and LBW-T0.8 piglets. In agreement with our data, dietary Trp supplementation reduced the serum lipids in rats [23] and the hepatic lipids in laying hens and broilers [24,25]. The data on the cellular lipid metabolism in LBW neonates fed with diets supplemented with higher amounts of Trp are scarce. In particular, there is a paucity of data on lipogenesis and lipolysis in LBW neonates fed with diets enriched in Trp. Here, we report a reduced hepatic mRNA abundance of ACC in both LBW-T0.4 and LBW-T0.8 and increased hepatic mRNA abundance of HADH in LBW-T0.4 piglets. ACC is a key enzyme involved in the biosynthesis of lipids, while HADH catalyzes the oxidation of fatty acids during beta oxidation. The downregulation of ACC and upregulation of HADH in the liver suggest that Trp supplementation reduces the lipogenesis but increases the lipolysis in the liver of LBW neonates, which may explain the reduced blood triglycerides. In support of our results, others showed that Trp increased the activity of CPT1 and oxidation of fatty acids [77]. They reported that Trp is involved in lipid metabolism through, serving as a precursor in the synthesis of melatonin [78,79]. Further research is warranted to better understand the role of endocrine signals that trigger alterations in lipid and glucose metabolism in LBW neonates receiving Trp-enriched diets. Given the role of Trp and its metabolism as one of the key modulators of gut microbiota [80] and also the importance of microbiota in glucose and lipid metabolism [81,82], it remains to be determined whether the gut microbiome is involved in alterations in glucose and lipid metabolism following Trp supplementation. Further, there appears to be a minor link between Trp metabolism metabolites and iron metabolism biomarkers [83]. Indicators of tryptophan metabolism have been shown to be positively correlated with hemoglobin and markers of iron metabolism [83]. Whether Trp-induced changes in iron metabolism play a role in the metabolic outcome of Trp-enriched diets requires further investigation.

While the role of brain serotonin, as an anorexigenic hormone in the regulation of feeding behavior, is documented [27,28,29,30,84], little is known of the role of peripheral serotonin in appetite regulation. In the present study, blocking 5-HT3 receptors with ondansetron reduced the feed intake in all groups. This was in line with a previous report, where ondansetron reduced the feed intake in rats [85]. Further investigation is needed to determine whether 5-HT3 receptors and peripheral serotonin are involved in the regulation of ingestive behavior in LBW piglets. More specifically, ondansetron decreased the intake transiently in LBW-T0, but that reduced the feed intake throughout the day in the LBW-T0.8 and NBW-T0 groups. This is indicative of a higher serotonergic signaling in the LBW-T0.8 and NBW-T0 groups, but a lower serotonergic tone in LBW-T0 piglets. The gastrointestinal tract is the main site for the conversion of Trp into serotonin [27,30,86], and Trp availability has been shown to enhance serotonin synthesis through the hydroxylation of Trp in a rate-limiting step by the action of tryptophan hydroxylase [87]. Therefore, our data suggest a higher availability of Trp in the LBW-T0.8 and NBW-T0 groups for the synthesis of gut-derived serotonin. In support of our data, a lower serotonin concentration in LBW piglets has been reported [27]. Since >95% of serotonin is produced in the gastrointestinal tract [27,30,86], the lower blood serotonin is suggestive of a reduced serotonin synthesis in the intestinal enterochromaffin cells of LBW piglets. Since the synthesized serotonin in enterochromaffin cells is stored in platelets and released after stimulation [88], further research is required to understand whether a lower peripheral serotonin is related with a higher rate of thrombocytopenia in LBW neonates [89].

## 5. Conclusions

To our knowledge, this is the first study assessing the effect of dietary Trp on glucose and lipid metabolism in LBW neonates. We demonstrated that supplemental Trp improves the glucose and lipid metabolism in an LBW piglet model through reducing hepatic lipogenesis, gluconeogenesis, and glucose efflux and increasing lipolysis and glycolysis. Further, we provided evidence of the role of peripheral serotonin in the regulation of feeding behavior and a lower serotonergic signaling in LBW piglets receiving no Trp supplement. Further research on the endocrine regulation of metabolic pathways involved in glucose and lipid metabolism following the administration of Trp in LBW neonates is required.

## Figures and Tables

**Table 1:** Effect of dietary L-tryptophan supplementation on the growth and feed efficiency of low-birthweight neonatal pigs.

Measurements	Diets ^1^				SEM ^2^	*p*-Value
	NBW-T0	LBW-T0	LBW-T0.4	LBW-T0.8		
Initial BW ^3^, g	4393 *	2853	2783	3123	145	<0.01
Final BW, g	14,172 *	10,877	10,634	10,818	335	<0.01
ADG ^3^, g/day	543 *	445	436	427	12	<0.01
ADMI ^3^, g/day	554 *	422	421	399	14	<0.01
ADPI ^3^, g/day	119 *	94	99	83 *	2.7	<0.01
G:F ^3^, g/g	1.00	1.05	0.97	1.07	0.01	0.26
G:P ^3^, g/g	4.65	4.77	4.30	5.14	0.10	0.02
Heart girth, cm	55.12 *	50.00	50.42	51.37	0.53	<0.01
Wither height, cm	34.75 *	32.00	31.14	31.25	0.46	<0.01
Body length, cm	54.00 *	48.62	46.42	47.37	0.66	<0.01

^1^ NBW-T0, normal-birthweight piglets fed a basal diet without supplemented L-tryptophan (Trp); LBW-T0, low-birthweight piglets fed a basal diet without supplemented Trp; LBW-T0.4, low-birthweight piglets fed a basal diet supplemented with 0.4% Trp; and LBW-T0.8, low-birthweight piglets fed a basal diet supplemented with 0.8% Trp. The values are the means. n = 8 for NBW-T0, LBW-T0, and LBW-T0.8, and n = 7 for LBW-T0.4. ^2^ SEM: standard errors of means. ^3^ BW: body weight; ADG: average daily gain; ADMI: average dry matter intake; ADPI: average daily protein intake; G:F: gain:feed; G:P: gain:protein. * *p* ≤ 0.05 vs. LBW-T0.

**Table 2:** Effect of dietary L-tryptophan supplementation on the intestinal morphology of low-birthweight neonatal pigs.

Measurements	Diets ^1^				SEM ^2^	*p*-Value
	NBW-T0	LBW-T0	LBW-T0.4	LBW-T0.8		
**Duodenum**						
Villi height, µm	562	497	585	530	20	0.44
Villi width, µm	138	124	138	123	3	0.13
Crypt depth, µm	229	224	230	198	8	0.54
Crypt width, µm	64	55	60	55	2	0.12
Muscle thickness, µm	385	376	447	386	17	0.46
Villi height:Crypt depth, µm:µm	2.90 ^#^	2.03	2.57	2.69	0.13	0.13
**Jejunum**						
Villi height, µm	619	595	589	594	28	0.98
Villi width, µm	121	106	118	125	3	0.23
Crypt depth, µm	200	211	221	256 ^#^	8	0.02
Crypt width, µm	48	49	53	55	1	0.24
Muscle thickness, µm	323	367	410	463	22	0.12
Villi height:Crypt depth, µm:µm	2.82	3.03	2.65	2.50	0.12	0.50

^1^ NBW-T0, normal-birthweight piglets fed a basal diet without supplemented L-tryptophan (Trp); LBW-T0, low-birthweight piglets fed a basal diet without supplemented Trp; LBW-T0.4, low-birthweight piglets fed a diet supplemented with 0.4% Trp; and LBW-T0.8, low-birthweight piglets fed a diet supplemented with 0.8% Trp. The values are the means. n = 4–5 for NBW-T0, LBW-T0.4, and LBW-T0.8, and n = 3–5 for LBW-T0. ^2^ SEM: standard errors of means. ^#^ 0.05 < *p* ≤ 0.1 vs. LBW-T0.

## Data Availability

All relevant data are listed in the manuscript.

## Supplementary Materials

The following are available online at https://www.mdpi.com/article/10.3390/nu13082561/s1, Figure S1: A representative screenshot of a thermal image; Figure S2: Full-length immunoblots from data shown in Figure 8; Table S1: Diet ingredients and calculated chemical composition (As-fed basis); Table S2: Chemical composition of the supplemental amino acids and whey powder used in the diets (As-fed basis); Table S3: Amino acid profile and crude protein of the whey protein concentrate used in the diets (As-fed basis); Table S4: Analyzed chemical composition of the diets (As-fed basis); Table S5: The sequences, location on template, amplicon size (bp), and GenBank accession numbers for primers used for reverse transcription quantitative real-time polymerase chain re-action (RT-qPCR); Table S6: The host, dilution, and supplier of primary and secondary antibodies for immunoblotting.
