# Supplementary material for: Dietary Tryptophan Supplementation Alters Fat and Glucose Metabolism in a Low-Birthweight Piglet Model
Source: Nutrients. 2021 Jul 26;13(8):2561. doi: 10.3390/nu13082561 (PMC8399558; doi:10.3390/nu13082561)
Supplement: Supplementary file 1 [file nutrients-13-02561-s001.zip › nutrients-1269349-supplementary.pdf]

Dietary Tryptophan Supplementation Alters Fat and Glucose Metabolism in a Piglet Model of Low Birthweight

Parniyan Goodarzi[1], Mohammad Habibi[1], Kennedy Roberts[1], Julia Sutton[1], Cedrick N. Shili[1], Dingbo Lin[2] and Adel Pezeshki[1]*

[1] Department of Animal and Food Sciences, Oklahoma State University, Stillwater, OK 74078, USA; parniyan.goodarzi@okstate.edu; mohammad.habibi@okstate.edu; kennedy.roberts@okstate.edu; julia.sutton@okstate.edu; cedrick.shili@okstate.edu; adel.pezeshki@okstate.edu
[2] Department of Nutritional Sciences, Oklahoma State University, Stillwater, OK 74078, USA; dingbo.lin@okstate.edu
* Correspondence: adel.pezeshki@okstate.edu; Tel.: (405) 744-8852

Supplementary Table S1. Diets ingredients and calculated chemical composition (As-fed basis)

**Ingredients %**	**Diets[1]**		
	**T0**	**T0.4**	**T0.8**
Whey powder[2]	52.92	52.92	52.92
Whey protein concentrate 36.17%[2]	24.58	24.58	24.58
Corn oil[2]	12.00	12.00	12.00
Sodium caseinate[2]	5.01	5.01	5.01
Dextrose[2]	1.02	0.97	0.91
Lactose[2]	1.00	1.00	1.00
Dicalcium phosphate 18.5%[2]	1.00	1.00	1.00
L-Alanin[2]	0.87	0.52	0.18
L-Tryptophan[2]	0.00	0.40	0.80
Limestone[2]	0.43	0.43	0.43
L-Lysine sulphate[2]	0.07	0.07	0.07
DL-Methionine[2]	0.12	0.12	0.12
L-Arginine[2]	0.21	0.21	0.21
L-Phenylalanine[2]	0.29	0.29	0.29
L-Histidine[2]	0.11	0.11	0.11
Vitamin premix[3]	0.25	0.25	0.25
Mineral premix[4]	0.12	0.12	0.12
**Calculated Chemical Composition[5]**			
Dry matter, %	95.64	95.64	95.65
ME, Mcal/kg	4.37	4.37	4.37
Crude protein, %	22.60	22.60	22.60
Crude fat, %	13.50	13.50	13.50
Lactose, %	51.34	51.34	51.34
SID Lysine, %	1.57	1.57	1.57
SID Threonine, %	1.00	1.00	1.00
SID Methionine, %	0.48	0.48	0.48
SID Tryptophan, %	0.31	0.71	1.11
SID Isoleucine, %	1.08	1.08	1.08
SID Valine, %	1.05	1.05	1.05
SID Arginine, %	0.65	0.65	0.65
SID Histidine, %	0.50	0.50	0.50
SID Lucine, %	1.83	1.83	1.83
SID Phenylalanine, %	0.94	0.94	0.94
SID Phe + Tyrosine, %	1.48	1.48	1.48
Calcium, %	0.90	0.90	0.90
Total phosphorus, %	0.73	0.73	0.73
Potassium, %	1.45	1.45	1.45

[1]T0: basal diet without supplemented L-tryptophan (Trp); T0.4: basal diet supplemented with 0.4% Trp; T0.8: basal diet supplemented with 0.8% Trp

[2]Whey powder, whey protein concentrate, corn oil, dextrose, lactose, dicalcium phosphate, limestone and L-arginine were obtained from Nutra Blend, LLC (Neosho, MO). Sodium caseinate was obtained from AMCO PROTEIN (Burlington, NJ). DL-methionine (99%) (MetAMINO®) and L-lysine (Biolys®) were obtained from Evonik (Kennesaw, GA). L-tryptophan (98%) was purchased from Ajinomoto (Overland Park, KS). L-valine

(96.5%), L-histidine, L-phenylalanine and L-alanine was obtained from Ajinomoto Health & Nutrition North America, Inc. (Raleigh, NC)

[3]Vitamin premix were purchased from Nutra Blend, LLC (Neosho, MO). Vitamin premix (per kg) contained: vitamin A, 1,650,000 IU; vitamin D_3, 660,000 IU; vitamin E, 17,600 IU; vitamin K, 1,320 mg; vitamin B12, 13.2 mg; niacin, 19,800 mg; pantothenic acid, 11,000 mg; riboflavin, 3,300 mg; phytase, 299,376 FYT

[4]Mineral premix were purchased from Nutra Blend, LLC (Neosho, MO). Mineral premix contained: copper, 11,000 ppm; iodine, 198 ppm; iron, 73,000 ppm; manganese, 22,000 ppm; selenium, 198 ppm; zinc, 73,000.

[5]National Swine Nutrition Guide (Version 2.1 Metric, ©2012 U.S. Pork Center of Excellence)

Supplementary Table S2. Chemical composition of supplemental amino acids and whey powder used in diets (As-fed basis)

**Chemical Composition**	**Ingredients [1]**			
	Arg	His	Phe	Whey powder
Dry matter, %	99.6	99.5	99.6	94.4
Crude protein, %	202.1	170.7	52.9	13.2
Crude fiber, %	<1.0	<1.0	<1.0	<1.0
Calcium, %	0.02	0.01	0.07	0.60
Phosphorus, %	0.01	0.01	<0.01	0.62

[1]The "less than" symbol (<) indicates the result was lower than the reporting limit for this laboratory ServiTech Laboratories (Dodge City, KS).

Supplementary Table S3. Amino acid profile and crude protein of whey protein concentrate used in diets (As-fed basis)

Items	Whey Protein Concentrate
Taurine*,%	0.15
Aspartic acid, %	3.82
Threonine, %	2.45
Serine, %	1.44
Glutamic acid, %	6.23
Proline, %	2.13
Glycine, %	0.68
Alanine, %	1.89
Cysteine, %	0.86
Valine, %	2.20
Methionine, %	0.67
Isoleucine, %	2.46
Leucine, %	3.89
Tyrosine, %	0.89
Phenylalanine, %	1.21
Hydroxylysine, %	0.02
Ornithine*, %	0.06
Lysine, %	3.33
Histidine, %	0.68
Arginine, %	0.92
Tryptophan, %	0.74
Crude protein#, %	36.17

* Non-proteinogenic amino acids

Crude protein= %N×6.25

Supplementary Table S4. Analyzed chemical composition of diets (As-fed basis)

Items	Diets[1]		
	T0	T0.4	T0.8
Taurine[2],%	0.13	0.12	0.12
Aspartic acid,%	1.84	1.88	1.87
Threonine,%	1.16	1.19	1.18
Serine,%	0.88	0.92	0.90
Glutamic acid,%	3.36	3.43	3.38
Proline,%	1.33	1.35	1.32
Lanthionine[2]	0.12	0.11	0.15
Glycine,%	0.39	0.40	0.40
Alanine,%	1.46	1.30	1.09
Cysteine,%	0.38	0.37	0.37
Valine,%	1.20	1.22	1.21
Methionine,%	0.45	0.56	0.47
Isoleucine,%	1.16	1.18	1.17
Leucine,%	1.98	2.03	2.01
Tyrosine,%	0.64	0.67	0.64
Phenylalanine,%	0.80	0.95	0.97
Hydroxylysine,%	0.04	0.04	0.02
Ornithine[2],%	0.01	0.01	0.01
Lysine,%	1.76	1.71	1.70
Histidine,%	0.49	0.51	0.52
Arginine,%	0.80	0.84	0.61
Tryptophan,%	0.36	0.84	1.29
Dry matter, %	95.3	95.8	95.0
Crude protein[3],%	21.5	21.4	20.7
Crude fiber, %	<1.0	<1.0	<1.0
Calcium, %	0.96	0.91	0.97
Phosphorus, %	0.72	0.67	0.71

[1]T0: basal diet without supplemented L-tryptophan (Trp); T0.4: basal diet supplemented with 0.4% Trp; T0.8: basal diet supplemented with 0.8% Trp. The diets were analyzed by Agricultural Experiment Station Chemical Laboratories (Columbia, MO) and ServiTech Laboratories (Dodge City, KS). The symbol "<" indicates that the values were lower than the reporting limit for the laboratory

[2]Non-proteinogenic amino acids

[3]Crude protein= %N×6.25

Supplementary Table S5. The sequences [forward (F) and reveres (R)], location on template, amplicon size (bp), and GenBank accession numbers for primers used for reverse transcription quantitative real-time polymerase chain reaction (RT-qPCR)

Genes[1]	Sequence (5′ → 3′)	Location on template	Amplicon length (bp)	GenBank accession no.
FAS	**F:** CTGCTGAAGCCTAACTCCTCG<br>**R:** TTGCTCCTTGGAACCGTCTG	584 - 604<br>771 - 790	207	NM_001099930.1
ACC	**F:** ATGTTTCGGCAGTCCCTGAT<br>**R:** TGTGGACCAGCTGACCTTGA	4870 - 4889<br>4983 - 5002	133	NM_001114269.1
HSL	**F:** GCTCCCATCGTCAAGAATC<br>**R:** TAAAGCGAATGCGGTCC	2043 - 2061<br>2291 - 2307	265	NM_214315.3
PPARα	**F:** CATCCTCGCGGGAAAGG<br>**R:**GGCCATACACAGTGTCTCCATGT	722 - 738<br>769 - 791	70	NM_001044526.1
SREBP-1	F: CGGACGGCTCACAATGC<br>R: GACGGCGGATTTATTCAGCTT	986 - 1002<br>1079 - 1099	114	NM_214157.1
HADH	**F:** GCCATCGTGGAGAACCTGAA<br>**R:** GAAATGGAGCCCGGCAAATC	461 - 480<br>600 - 619	159	NM_214331.1
PGC1α	**F:** GATGTGTCGCCTTCTTGTTC<br>**R:** CATCCTTTGGGGTCTTTGAG	1629 - 1648<br>1702 - 1721	93	NM_213963.2
LPL	**F:** CCCTATACAAGAGGGAACCGGAT<br>**R:** CCGCCATCCAGTCGATAAACGT	448 - 470<br>564 - 580	138	NM_214286.1
CD36	**F:** CTGGTGCTGTCATTGGAGCAGT<br>**R:** CTGTCTGTAAACTTCCGTGCCTGTT	443 - 464<br>579 - 603	161	NM_001044622.1
GCK	**F:** CCGACTTCCTGGACAAGCAT<br>**R:** ATCGTGGCCACAGTGTCATT	1040 -1059<br>1258 -1277	238	XM_003134883.2
PFKL	**F:** ACTCCCTTCGACCGGAACTA<br>**R:** TGCTCAAAGTCGGTGTCCTC	2119 - 2138<br>2296 - 2315	197	XM_021071510.1
GLUT2	**F:** GGTTCATGGTGGCCGAGTT<br>**R:** ATTGCGGGTCCAGTTGC	1260 - 1278<br>1326 - 1342	83	NM_001097417.1
PEPCK	**F:** CTGGGAAGGCATTGATCAGC<br>**R:** AGCGAGAGTTAGGATGTACA	1336 - 1335<br>1426 - 1445	110	NM_001161753.1
G6PC	**F:** TGAACGTCTGTCTGTCACGA<br>**R:** ATACTTCTTGAGGCTGGCGT	491 - 510<br>608 - 627	137	NM_001113445.1
GLUT1	**F:** GGAGATGAAGGAGGAGAGCC<br>**R:** TAGAAAACCGCGTTGATGCC	982 - 1001<br>1112 - 1131	150	XM_021096908.1
PC	**F:** GGACTTCACTGCCACCTTTG<br>**R:** GCTCCACCTCAAACTCCTCT	3063 - 3082<br>3135 - 3154	92	NM_214349.1
PKLR	**F:** CCCACTGAAGTCACCGCTAT<br>**R:** GAGGAAGCCACGGAGTTTT	1357 – 1376<br>1626 - 1644	288	XM_021089721.1
β-Actin	**F:** CTGCGGCATCCACGAAACT<br>**R:** AGGGCCGTGATCTCCTTCTG	944 - 962<br>1071 - 1090	147	XM_003124280.5

[1] FAS = fatty acid synthase; ACC = acetyl-CoA carboxylase; HSL = Hormone-sensitive lipase; PPARα = Peroxisome proliferator activated receptor alpha; SREBP-1 = sterol regulatory element binding transcription factor 1; HADH = Hydroxyacyl-CoA dehydrogenase; PGC1α = PPARG coactivator 1 alpha; LPL = lipoprotein lipase; CD36 = cluster of differentiation 36 molecule, GCK = glucokinase; PFKL = phosphofructokinase, liver type; GLUT2 = glucose transporter 2; PEPCK = phosphoenolpyruvate carboxykinase; G6PC = glucose-6-phosphatase catalytic; GLUT1 = glucose transporter 1; PC = pyruvate carboxylase; PKLR = pyruvate kinase, liver and RBC .

Supplementary Table S6. The host, dilution and supplier of primary and secondary antibodies for immunoblotting.

Antibodies	Host	Dilution	Vendor
Anti-Tryptophan hydroxylase 2 (TPH2)	Goat	1:500	Abcam, Cambridge, MA, #ab121013
Anti-Sodium-glucose co-transporter 1 (SGLT-1)	Rabbit	1:2000	Thermo Scientific, Rockford, IL, #PA5-28240
Anti-Carnitine palmitoyltransferase I α (CPT1α)	Rabbit	1:3000	Thermo Scientific, Rockford, IL, #15184-1-AP
Anti-Glucose transporter 2 (GLUT-2)	Rabbit	1:500	Thermo Scientific, Rockford, IL, #720238
Anti-GAPDH [6C5] (HRP)	Mouse	1:5000	Abcam, Cambridge, MA, #ab105428
Anti-Goat IgG H&L (HRP)	Donkey	1:10000	Abcam, Cambridge, MA, #ab205723
Anti-Rabbit IgG H&L (HRP)	Goat	1:1600	Abcam, Cambridge, MA, #ab205718

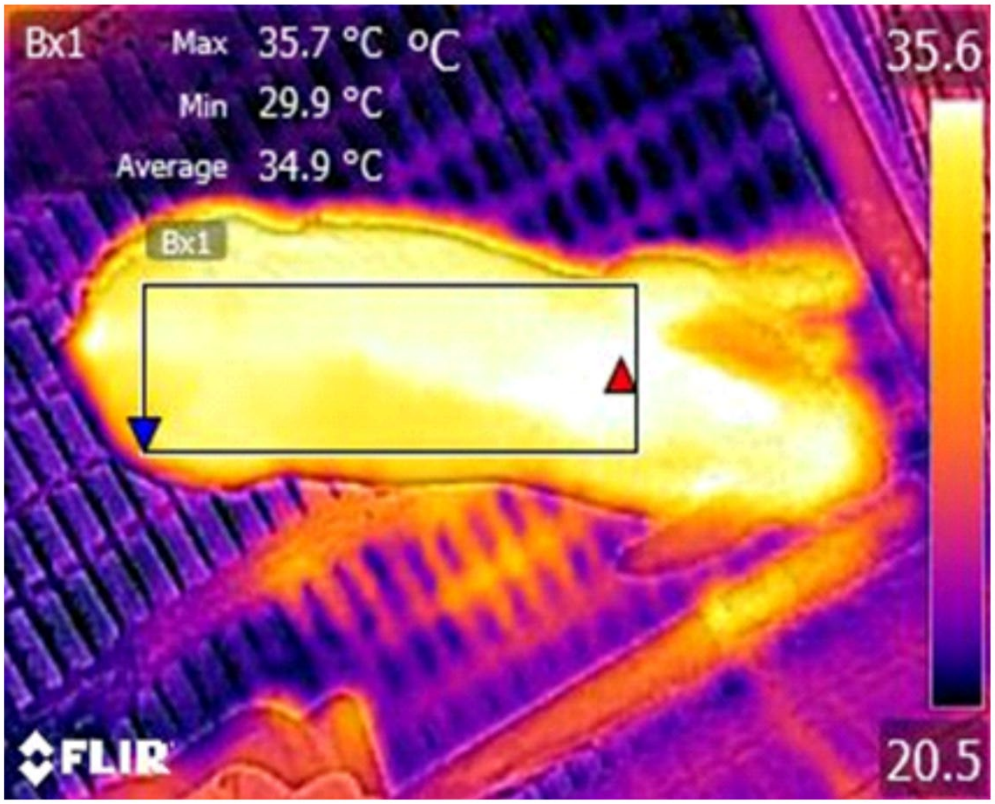

Supplementary Figure S1. A representative screenshot of a thermal image. Dorsal surface body mean temperature was obtained by drawing a rectangular in the entire back of piglets approximately from shoulders to the rump of the animal using a rectangular drawing tool of FLIR camera software (FLIR Research Studio, Wilsonville, OR).

A1

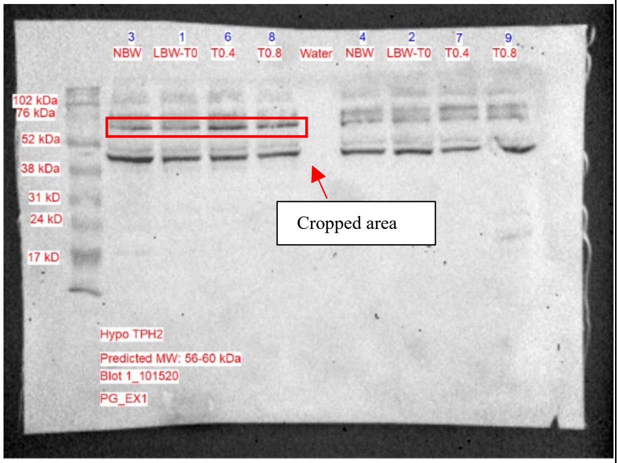
3 NBW
1 LBW-T0
6 T0.4
8 T0.8
Water
4 NBW
2 LBW-T0
7 T0.4
9 T0.8
102 kDa
76 kDa
52 kDa
38 kDa
31 kD
24 kD
17 kD
Cropped area
Hypo TPH2
Predicted MW: 56-60 kDa
Blot 1_101520
PG_EX1

A2

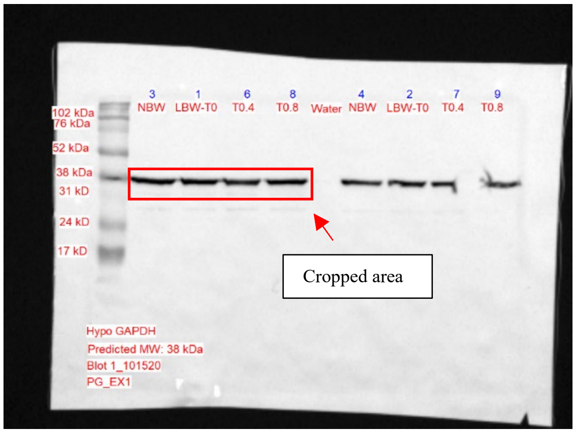
3 NBW
1 LBW-T0
6 T0.4
8 T0.8
Water
4 NBW
2 LBW-T0
7 T0.4
9 T0.8
102 kDa
76 kDa
52 kDa
38 kDa
31 kD
24 kD
17 kD
Cropped area
Hypo GAPDH
Predicted MW: 38 kDa
Blot 1_101520
PG_EX1

B1

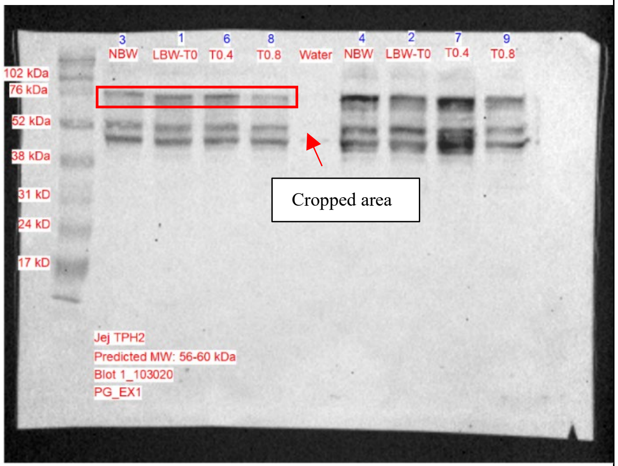
3 NBW
1 LBW-T0
6 T0.4
8 T0.8
Water
4 NBW
2 LBW-T0
7 T0.4
9 T0.8
102 kDa
76 kDa
52 kDa
38 kDa
31 kD
24 kD
17 kD
Cropped area
Jej TPH2
Predicted MW: 56-60 kDa
Blot 1_103020
PG_EX1

B2

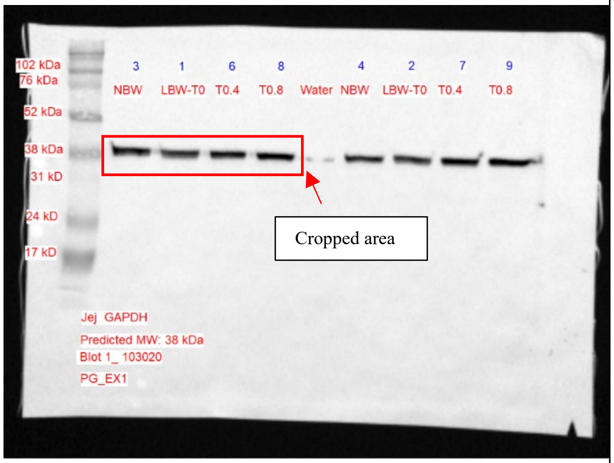
3 NBW
1 LBW-T0
6 T0.4
8 T0.8
Water
4 NBW
2 LBW-T0
7 T0.4
9 T0.8
102 kDa
76 kDa
52 kDa
38 kDa
31 kD
24 kD
17 kD
Cropped area
Jej GAPDH
Predicted MW: 38 kDa
Blot 1_ 103020
PG_EX1

C1

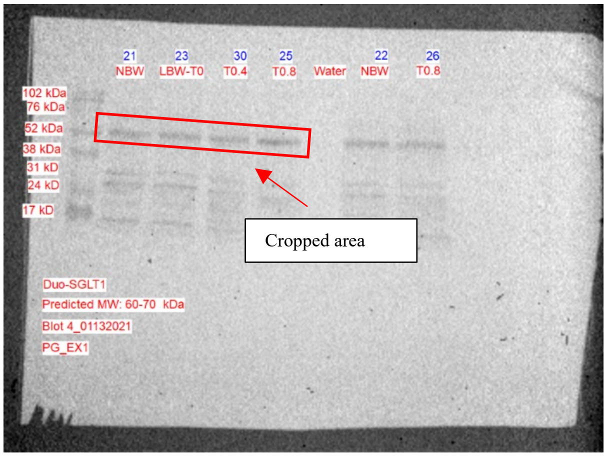
21 NBW
23 LBW-T0
30 T0.4
25 T0.8
Water
22 NBW
26 T0.8
102 kDa
76 kDa
52 kDa
38 kDa
31 kD
24 kD
17 kD
Cropped area
Duo-SGLT1
Predicted MW: 60-70 kDa
Blot 4_01132021
PG_EX1

C2

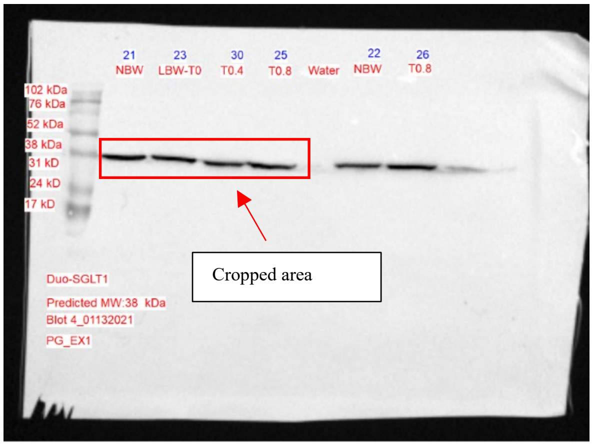
21 NBW
23 LBW-T0
30 T0.4
25 T0.8
Water
22 NBW
26 T0.8
102 kDa
76 kDa
52 kDa
38 kDa
31 kD
24 kD
17 kD
Cropped area
Duo-SGLT1
Predicted MW:38 kDa
Blot 4_01132021
PG_EX1

D1

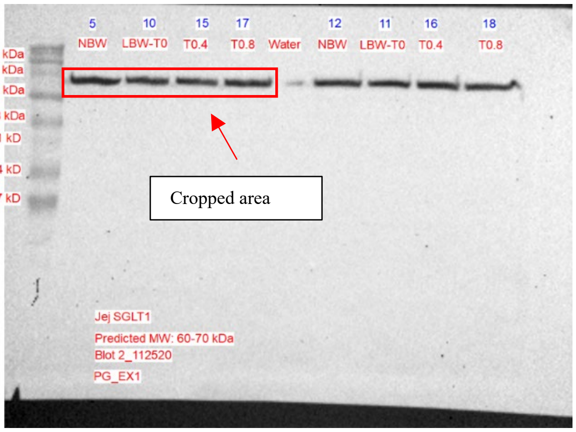
5 NBW
10 LBW-T0
15 T0.4
17 T0.8
Water
12 NBW
11 LBW-T0
16 T0.4
18 T0.8
102 kDa
76 kDa
52 kDa
38 kDa
31 kD
24 kD
17 kD
Cropped area
Jej SGLT1
Predicted MW: 60-70 kDa
Blot 2_112520
PG_EX1

D2

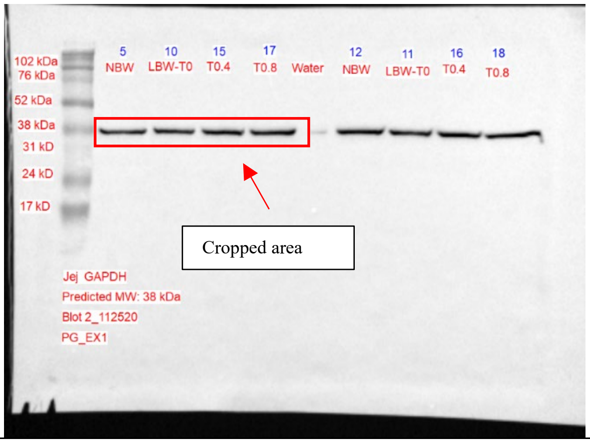
5 NBW
10 LBW-T0
15 T0.4
17 T0.8
Water
12 NBW
11 LBW-T0
16 T0.4
18 T0.8
102 kDa
76 kDa
52 kDa
38 kDa
31 kD
24 kD
17 kD
Cropped area
Jej GAPDH
Predicted MW: 38 kDa
Blot 2_112520
PG_EX1

E1

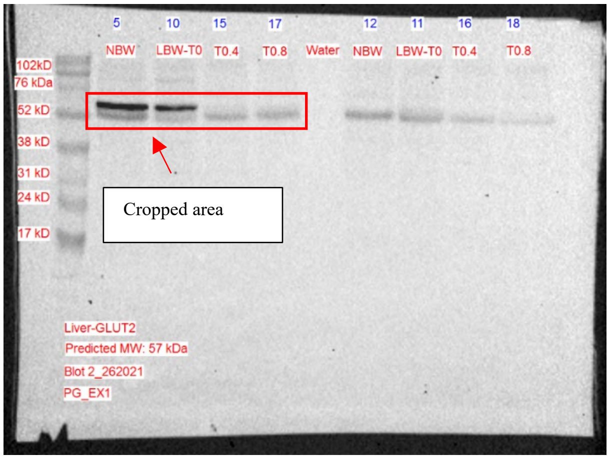
5 10 15 17 12 11 16 18
NBW LBW-T0 T0.4 T0.8 Water NBW LBW-T0 T0.4 T0.8
102kD
76 kDa
52 kD
38 kD
31 kD
24 kD
17 kD
Cropped area
Liver-GLUT2
Predicted MW: 57 kDa
Blot 2_262021
PG_EX1

E2

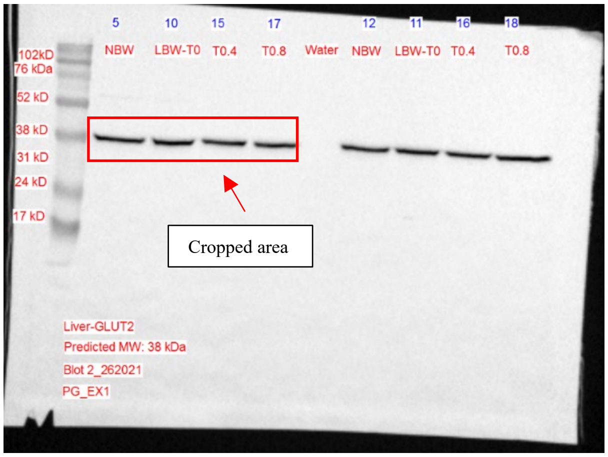
5 10 15 17 12 11 16 18
NBW LBW-T0 T0.4 T0.8 Water NBW LBW-T0 T0.4 T0.8
102kD
76 kDa
52 kD
38 kD
31 kD
24 kD
17 kD
Cropped area
Liver-GLUT2
Predicted MW: 38 kDa
Blot 2_262021
PG_EX1

F1

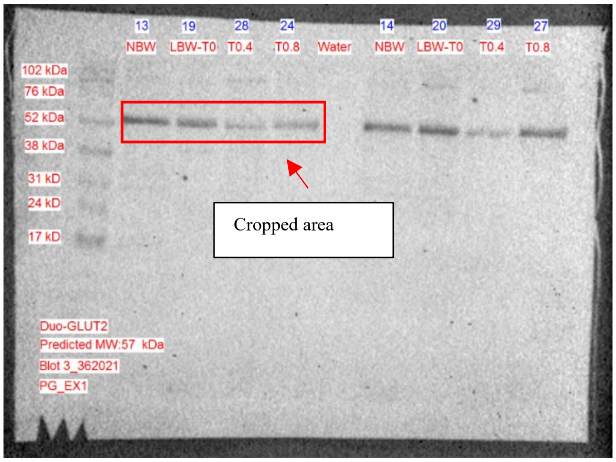
13 19 28 24 14 20 29 27
NBW LBW-T0 T0.4 T0.8 Water NBW LBW-T0 T0.4 T0.8
102 kDa
76 kDa
52 kDa
38 kDa
31 kD
24 kD
17 kD
Cropped area
Duo-GLUT2
Predicted MW:57 kDa
Blot 3_362021
PG_EX1

F2

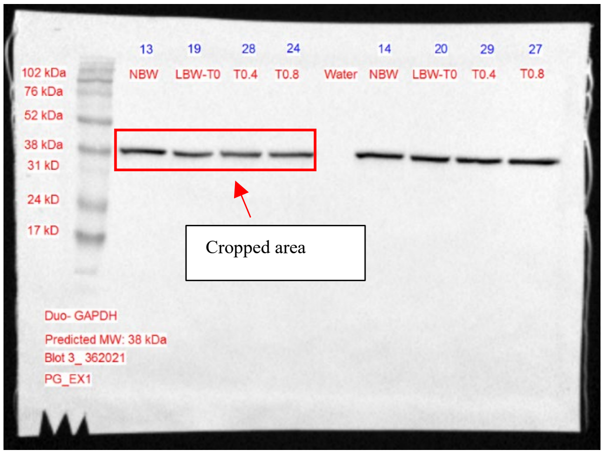
13 19 28 24 14 20 29 27
NBW LBW-T0 T0.4 T0.8 Water NBW LBW-T0 T0.4 T0.8
102 kDa
76 kDa
52 kDa
38 kDa
31 kD
24 kD
17 kD
Cropped area
Duo- GAPDH
Predicted MW: 38 kDa
Blot 3_ 362021
PG_EX1

G1

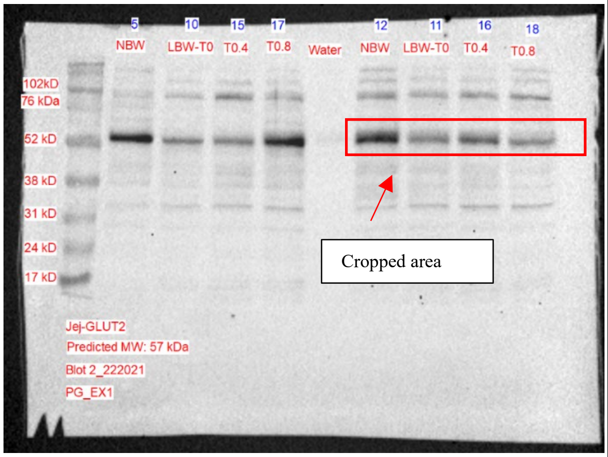
5 10 15 17 12 11 16 18
NBW LBW-T0 T0.4 T0.8 Water NBW LBW-T0 T0.4 T0.8
102kD
76 kDa
52 kD
38 kD
31 kD
24 kD
17 kD
Cropped area
Jej-GLUT2
Predicted MW: 57 kDa
Blot 2_222021
PG_EX1

G2

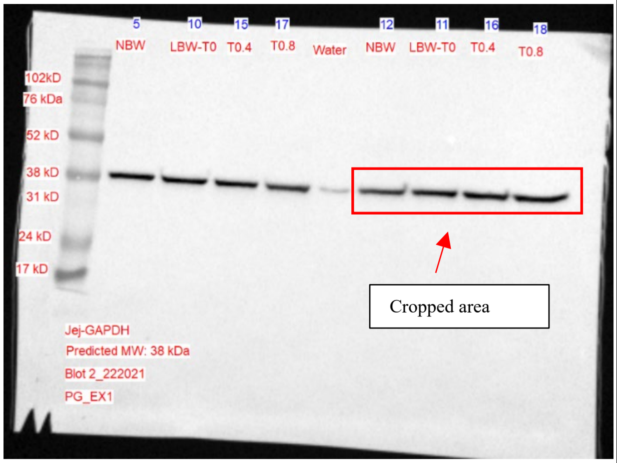
5 10 15 17 12 11 16 18
NBW LBW-T0 T0.4 T0.8 Water NBW LBW-T0 T0.4 T0.8
102kD
76 kDa
52 kD
38 kD
31 kD
24 kD
17 kD
Cropped area
Jej-GAPDH
Predicted MW: 38 kDa
Blot 2_222021
PG_EX1

H1

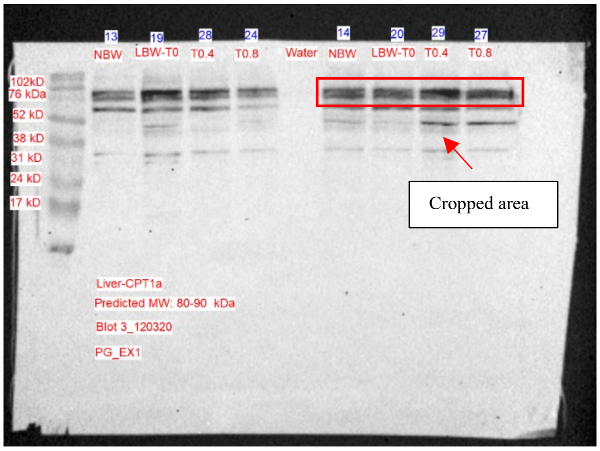
13 19 28 24 14 20 29 27
NBW LBW-T0 T0.4 T0.8 Water NBW LBW-T0 T0.4 T0.8
102kD
76 kDa
52 kD
38 kD
31 kD
24 kD
17 kD
Cropped area
Liver-CPT1a
Predicted MW: 80-90 kDa
Blot 3_120320
PG_EX1

H2

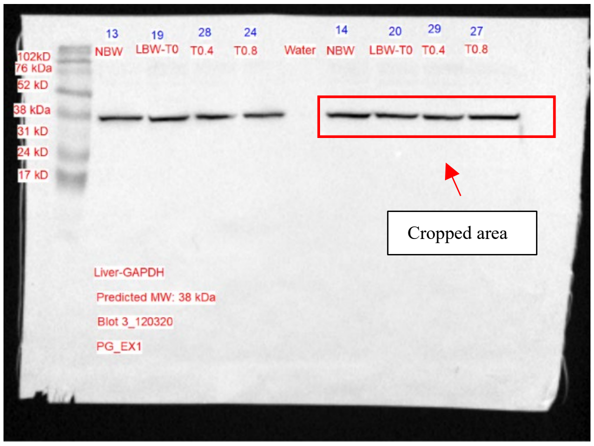
13 19 28 24 14 20 29 27
NBW LBW-T0 T0.4 T0.8 Water NBW LBW-T0 T0.4 T0.8
102kD
76 kDa
52 kD
38 kDa
31 kD
24 kD
17 kD
Cropped area
Liver-GAPDH
Predicted MW: 38 kDa
Blot 3_120320
PG_EX1

I1

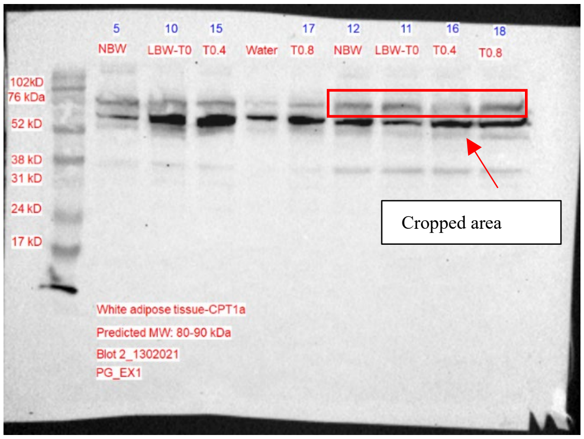

I2

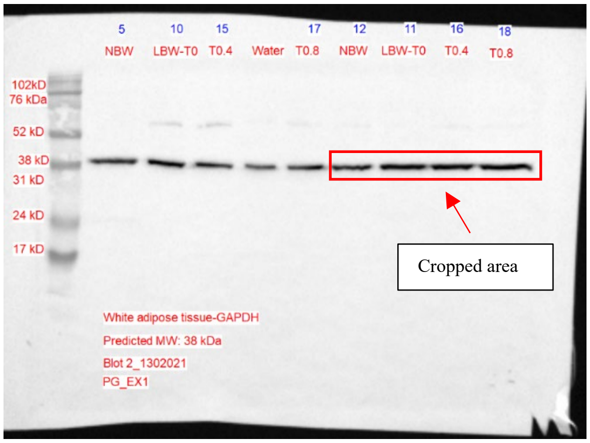

Supplementary Figure S2. Full-length immunoblots from data shown in Figure 8. (A1) anti-tryptophan hydroxylase 2 (TPH2) in hypothalamus, (A2) anti-GAPDH as a loading control for TPH2 in hypothalamus, (B1) anti-TPH2 in jejunum, (B2) anti-GAPDH as a loading control for TPH2 in jejunum, (C1) anti-sodium-glucose co-transporter 1 (SGLT-1) in duodenum, (C2) anti-GAPDH as a loading control for SGLT-1 in duodenum, (D1) anti-SGLT-1 in jejunum (D2) anti-GAPDH as a loading control for SGLT-1 in jejunum, (E1) anti-glucose transporter 2 (GLUT-2) in liver, (E2) anti-GAPDH as a loading control for GLUT-2 in liver, (F1) anti- GLUT-2 in duodenum, (F2) anti-GAPDH as a loading control for GLUT-2 in duodenum, (G1) anti-GLUT-2 in jejunum, (G2) anti-GAPDH as a loading control for GLUT-2 in jejunum, (H1) anti-carnitine palmitoyltransferase I α (CPT1α) in liver, (H2) anti-GAPDH as a loading control for CPT1α in liver, (I1) anti-CPT1α in white adipose tissue, (I2) anti-GAPDH as a loading control for CPT1α in white adipose tissue. NBW-T0, normal birthweight piglets fed a basal diet without supplemented L-tryptophan (Trp); LBW-T0, low birthweight piglets fed a basal diet without supplemented Trp; LBW-T0.4, low birthweight piglets fed a diet supplemented with 0.4% Trp; and LBW-T0.8, low birthweight piglets fed a diet supplemented with 0.8% Trp. n = 8 for NBW-T0, LBW-T0, LBW-T0.8 and n=7 for LBW-T0.4.
